# Bacterial endosymbiont *Cardinium* cSfur genome sequence provides insights for understanding the symbiotic relationship in *Sogatella furcifera* host

**DOI:** 10.1186/s12864-018-5078-y

**Published:** 2018-09-19

**Authors:** Zhen Zeng, Yating Fu, Dongyang Guo, Yuxuan Wu, Olugbenga Emmanuel Ajayi, Qingfa Wu

**Affiliations:** 1 Hefei National Laboratory for Physical Sciences at Microscale, University of Science and Technology of China, Hefei, 230027 China; 2 CAS Key Laboratory of Innate Immunity and Chronic Disease, University of Science and Technology of China, Hefei, 230027 China; 3 Department of Computer Science, University of Nottingham Ningbo China, Zhejiang, 315100 China

**Keywords:** Bacterial endosymbionts, *Cardinium*, Genomic analysis, *Sogatella furcifera*

## Abstract

**Background:**

*Sogatella furcifera* is a migratory pest that damages rice plants and causes severe economic losses. Due to its ability to annually migrate long distances, *S. furcifera* has emerged as a major pest of rice in several Asian countries. Symbiotic relationships of inherited bacteria with terrestrial arthropods have significant implications. The genus *Cardinium* is present in many types of arthropods, where it influences some host characteristics. We present a report of a newly identified strain of the bacterial endosymbiont *Cardinium* cSfur in *S. furcifera*.

**Result:**

From the whole genome of *S. furcifera* previously sequenced by our laboratory, we assembled the whole genome sequence of *Cardinium* cSfur. The sequence comprised 1,103,593 bp with a GC content of 39.2%. The phylogenetic tree of the Bacteroides phylum to which *Cardinium* cSfur belongs suggests that *Cardinium* cSfur is closely related to the other strains (*Cardinium* cBtQ1 and cEper1) that are members of the Amoebophilaceae family. Genome comparison between the host-dependent endosymbiont including *Cardinium cSfur* and free-living bacteria revealed that the endosymbiont has a smaller genome size and lower GC content, and has lost some genes related to metabolism because of its special environment, which is similar to the genome pattern observed in other insect symbionts. *Cardinium* cSfur has limited metabolic capability, which makes it less contributive to metabolic and biosynthetic processes in its host. From our findings, we inferred that, to compensate for its limited metabolic capability, *Cardinium* cSfur harbors a relatively high proportion of transport proteins, which might act as the hub between it and its host. With its acquisition of the whole operon related to biotin synthesis and glycolysis related genes through HGT event, *Cardinium* cSfur seems to be undergoing changes while establishing a symbiotic relationship with its host.

**Conclusion:**

A novel bacterial endosymbiont strain (*Cardinium* cSfur) has been discovered. A genomic analysis of the endosymbiont in *S. furcifera* suggests that its genome has undergone certain changes to facilitate its settlement in the host. The envisaged potential reproduction manipulative ability of the new endosymbiont strain in its *S. furcifera* host has vital implications in designing eco-friendly approaches to combat the insect pest.

**Electronic supplementary material:**

The online version of this article (10.1186/s12864-018-5078-y) contains supplementary material, which is available to authorized users.

## Background

The white-backed planthopper (WBPH), *Sogatella furcifera* (Horvath), is a small oligophytophagous insect that belongs to the order Hemiptera. The insect is a highly devastating pest of rice and damages rice by feeding directly on it. The attacked plants turn yellow and later acquire a rust-red appearance, spreading from the leaf tips to the rest of the plants. *S. furcifera* can become sufficiently numerous to kill the plants by hopper burn, where the tillers dry up and turn brown due to excessive removal of plant sap [1]. WBPH also serves as a vector for transmitting plant viruses such as southern rice black-streaked dwarf virus (SRBSDV). Due to their ability to annually migrate long distances, WBPH has emerged as a major pest of rice in several Asian countries [2].

It is now widely recognized that the symbiotic microorganisms of arthropods play a crucial role in the ecology and evolution of their hosts. Such endosymbionts are primarily transmitted vertically, that is, from mothers to their offspring. Insect endosymbionts have two broad categories; primary endosymbionts (P-endosymbionts) and secondary endosymbionts (S-endosymbionts) [3]. The P-endosymbionts form obligate associations and display co-speciation with their insect hosts [4], in that neither the bacteria nor the insect is viable without the other. These maternally inherited P-endosymbionts are perceived to help the host either by providing nutrients that the host cannot obtain itself or by metabolizing insect waste products into safer forms. For example, the putative primary role of *Buchnera* is to synthesize essential amino acids and supply its host *Acyrthosiphon pisum,* since the aphid cannot acquire essential amino acids from its natural diet of plant sap [5]. The S-endosymbionts are less understood, though not obligate symbionts, they exhibit a multifarious association with their hosts [3], and are sometimes horizontally transferred between hosts [6]. It was reported that the pea aphid (*Acythosiphon pisum*) contains at least three S-endosymbionts, viz., *Hamiltonella defensa*, *Regiella insecticola* and *Serratia symbiotica*. Notably, *Hamiltonella defensa* confers resistance to parasitoid wasps; *Regiella insecticola* can confer protection against fungal pathogens and *Serratia symbotica* helps the host aphid bear heat shock [7].

*Candidatus Cardinium* is a Gram-negative bacterium belonging to the phylum Cytophaga-Flavobacterium-Bacteroides (CFB) [8]. Symbionts belonging to the genus *Cardinium* are present in many types of arthropods including *Bemisia tabaci* [9], spider mites [10], *Culicoides* [11], and plant parasitic nematodes [12]. *Cardinium* has been reported as an S-endosymbiont in *Bemisia tabaci* [13, 14]. Thus far, the *Cardinium* infection rate in arthropods has been estimated as close to 7% [15]. The bacterial endosymbiont *Cardinium* present in arthropod species is capable of influencing host characteristics such as vector competence [16] and nutrient provision [17]. *Cardinium* reportedly causes cytoplasmic incompatibility (CI) in *Encarsia pergandiella* and spider mites [18] and has also been implicated in the feminization of *Brevipalpus californicus* [19]. The maternally inherited *Cardinium* was recently discovered to be a reproductive manipulator [20]. *Wolbachia*, a common intracellular bacterium found in arthropods and nematodes co-exists in the same host as *Cardinium* [21–23]. Of the three rice planthoppers (*Laodelphax striatellus*, *Nilaparvata lugens* and *Sogatella furcifera*), only *S. furcifera* is co-infected with both *Cardinium* and *Wolbachia*, while the other two are only infected with *Wolbachia.* Notably, the co-infection rates of the two endosymbionts in the *S. furcifera* host across different regions have been reported, with the highest being 60.9% while the lowest 26.1% [24]

Because of their unbalanced diet, many phloem-feeding insects develop symbiotic relationships with their endosymbionts, thus providing the hosts with nutrients [3]. Based on research [25] previously published by our group, which assembled and annotated the whole genome sequence and transcriptome of *S. furcifera*, we obtained and analyzed the whole genome sequence of a novel strain of the *Cardinium* endosymbiont cSfur in *S. furcifera*. A genome analysis revealed that the *Cardinium* cSfur genome has changed significantly to adapt to the symbiotic relationship with the *S. furcifera* host.

## Methods

### Genome assembly

A total of 241.3 Gb of raw reads from the whole genome sequencing of *S. furcifera* (PRINA331022) generated from 17 insert libraries ranging between 180 bp and 40 kbp were downloaded via GigaDB (http://gigadb.org/dataset/100255). The reads were trimmed by removing adapter sequences, and low-quality or N bases with Trimmomatic program [26] (with the settings: ILLUMINACLIP:adapter-seq-file:2:30:10 LEADING:3 TRAILING:3 SLIDINGWINDOW:4:15 MINLEN:36). The clean reads of both long and short-insert libraries were mapped onto the assembled genome sequences of *Sogetella furcifera*, mitochondria and *Wolbachia* symbiont [25] using Bowtie2 [27] with the default settings, and all unmapped reads were extracted for further assembly of endosymbiont genomes. The duplicated reads were removed before assembling using FastUniq [28]. The remaining short reads (insert size ≤680 bp) were assembled using SOAPdenovo2 [29], with kmer size of 67 and pair_num_cutoff of 5. The other 2 *Cardinium* genomes (*Cardinium* cEper1 and cBtQ1), obtained from the NCBI (National Center for Biotechnology Information) with project accession numbers of PRJEA66241 and PRJEB4234, respectively, were used as references to search the assembled contigs using the BLASTN with a e-value cutoff of 1e^− 10^. All assembled contigs belonging to *Cardinium* were scaffolded with longer reads using SSPACE v2.0 BASIC [30], and the gaps were filled with short reads using GapFiller v1.9 [31] with the default settings.

### Genome annotation and analysis

The total chromosome ORFs of the previously sequenced *Cardinium* strains (cEper1 and cBtQ1) available in the NCBI were used to train Glimmer3.02 [32] and Prodigal.v2 [33] for the prediction of Open Reading Frames (ORFs). The NCBI non-redundant protein database (NR) was downloaded in Dec. 2016. The BLASTP program within the ncbi-blast-2.2.26 suite was thereafter used to further refine the protein-coding genes of the high confidence gene models predicted by Glimmer and Prodigal against the NR database (with the cutoffs: identity of 30%, e-value of 1e^− 5^ and coverage of 30%) according to the Common Gene Annotation Process [34]. The transfer RNA, rRNA and tmRNA were predicted with tRNAscan-SE [35], RNAmmer [36] and Aragorn [37] respectively. The gene annotation was mainly based on a homology search with NR, COG (2003), Pfam 29.0 [38] and TIGRfam 15.0 [39]. The resulting protein-coding genes were submitted to BLASTP against NR and COG (e-value: 1e^− 5^), while the Pfam and TIGRfam assignments were implemented by HMMER 3.0 [40]. For Pfam, the gathering threshold (−-cut_ga) was used, while for TIGRfam, the noise cutoff (−-cug_nc) was used [41]. TMHMM v.2.0 [42] was used to predict the transmembrane helices in proteins and the prediction of signal peptides was performed with SignalP 4. 1 [43]. Using the KEGG GENES database as the reference sequence set, KAAS [44] was used to identify the pathways, especially the metabolism pathway, in the *Cardinium* genome. IS elements were detected using the web server ISsaga [45]. BLASTP was used to find transport proteins against clustering TCDB (Transport Classification Database) [46] with cutoff values of 1e^− 10^ e-value, 70% sequence identity and 40% sequence coverage.

The repetitive regions of the 3 *Cardinium* genomes were plotted with NUCmer in MUMmer 3.0. The genomic sequence redundancy was estimated with the BLASTN program within the ncbi-blast-2.2.26 suite using only each chromosome genome (no plasmid was detected in the *Cardinium* of *S. furcifera*) as both the query and subject with an e-value cutoff of 1e^− 20^. The alignment with an identity over 95% was used to calculate the redundancy. The ANI calculator [47] was used to compute the average nucleotide identity between every two *Cardinium* genomes.

### Phylogenetic and Phylogenomic analyses

For the *Cardinium* phylogenomic reconstruction, 46 Bacteroides genomes and a non-Bacteroides species used as an outgroup were selected from the Microbial Genome Database (MGDB) [48], 16 orthologous single copy genes related to replication/recombination/repair, translation/ribosomal structure/biogenesis and post-translational modification/protein turnover were identified from the 47 genomes by the homology search tool of the MGDB (Additional file 1). The protein sequences were concatenated and aligned with MAFFT v7.158b (L-INS-i) [49], and then refined with Gblocks to prune the alignment and retain the conserved blocks [50]. The top BLASTP hits of the amino acid sequence for gene gyrB were selected for alignment with MAFFT v7.158b (L-INS-i) [49]. The phylogenetic trees of both species and gyrB gene were reconstructed using MEGA6 [51] under the ML criterion with 1000 bootstrap replicates.

### Horizontal gene transfer analysis

The putative genes acquired by horizontal gene transfer were first predicted by two methods; one was based on a homology search while the other was based on the GC content of the genes [52]. The *Cardinium* proteins were searched against the NR database in NCBI using BLASTP (with the cutoffs: identity of 50%, e-value of 1e^− 5^ and coverage of 70%). The genes were considered to be the candidates acquired by HGT event if none of their top 10 hits (excluding genes of the other two *Cardinium* strains) was from organisms belonging to the Bacteroides. Thereafter, the G + C (1), G + C (2), G + C (3) and G + C (T) (the G + C contents of codon positions 1, 2, 3 and the total G + C content) of every gene were calculated. Because shorter genes are more likely to be extraneous, genes of less than 300 bp in length were excluded when the mean values and standard deviation (δ) were calculated. The genes are considered extraneous if their G + C (T) content deviates by more than 1.5δ [53] from the mean value or if the deviations of G + C (1) and G + C (3) are of the same sign, and at least one was greater than 1.5δ. The genes identified using both selection methods were considered as candidates acquired by horizontal gene transfer event. For further identification, each of the candidate genes, together with their respective best 50 BLASTP hits, were aligned with MAFFT v7.158b (L-INS-i) [49], after which the phylogenetic trees were reconstructed with MEGA6 software [51] (Additional file 2) to ascertain their involvement in the unexpected phylogenetic tree topology. The nearest neighbors of the genes acquired by the event of HGT were identified by the least number of nodes in the tree. The genes that have at least one orthologous gene in the other two *Cardinium* genomes were defined as genes involved in an ancient HGT event, and those without orthologous genes in the other two *Cardinium* genomes were defined as being acquired by recent events.

### Comparative genome analyses of *Cardinium* cSfur

A statistical comparative analysis was performed to further elucidate the difference between the host-dependent and free-living bacteria using 17 genomes from the order Cytophagales, including three *Cardinium* genomes. BLASTClust [54] was initially used to cluster each genome, and thereafter, COG categories were assigned for each cluster with cutoffs of a 70% alignment match and an e-value of 1e^− 5^. Afterwards, the phylogenetic profiles of the 17 genomes were determined with respect to their gene cluster COGs. The relative percentages of each COG category in each bacterium were used for hierarchical clustering and plotted with pheatmap [55]. Differences in the relative percentages of each COG category between host-dependent and free-living bacteria were evaluated with non-parametric Wilcoxon test [56].

The orthologous genes among *Cardinium* cEper1, cBtQ1 and cSfur were identified using BLASTClust with a cutoff alignment coverage of 70% [57] and identity of 50%. A circle plot of the three *Cardinium* genomes was constructed with MCscan software [58] using only the chromosome. The syntenic segments between *Cardinium* cSfur genome and the plasmid sequences (pCher and pCHV of other two *Cardinium* strains) were identified with BLASTP and manual parsing.

### Extraction of bacterial DNA and PCR verification

To verify the speculation that *Cardinium* cSfur is not confined to a special tissue of the host *S. furcifera*, a fragment of the *Cardinium* 16S rDNA gene (766 bp) and gyrB gene (575 bp) were used to detect the existence of *Cardinium* cSfur. Five female and 5 male adults of *S. furcifera* were collected respectively, and five tissues (MG: malpighian tubule, OV/TE: ovary/testis, SG: salivary gland, MT: midgut, FB: fat body) were dissected, and then the bacterial DNA was extracted from the sections of tissues and the rest of the body with the Ezup Column Bacteria Genomic DNA Purification Kit (Sangon Biotech, Shanghai, China). The primers of the 16S rDNA gene were 256f (5’-ACCGAGTGGTTCCGATGCTA-3′) and 1021r (5’-GTCCCGAAGGAACCCTCAAT-3′), and primers of the GyrB gene were 924f (5’-TATGCATGTCACTGGATTTAGAAGA-3′) and 1498r (5’-TCATATTCCTAACCTGCTCGTTATC-3′). The PCR program was: 95 °C for 2 min; 36 cycles of 95 °C for 30 s, 58 °C for 30 s, 75 °C for 45 s; 72 °C for 5 min; and 12 °C for 60 min.

## Results

### Genomic features of bacterial endosymbiont *Cardinium* cSfur

The genome of *Cardinium* cSfur comprises 1,103,593 base pairs (bp) with a 39.2% GC content. The coverage of the bacterial endosymbiont was approximately 120x. A genome annotation showed that 795 coding DNA sequences (CDS) with an average length of 1052 bp were detected (Table 1). The cSfur genome contains only one set of rRNA genes (5S, 16S and 23S rRNA). The 23S rRNA gene precedes the 5S rRNA, while the four CDSs and the 16S rRNA gene follow in respective orders. Additionally, the genome contains 35 tRNA genes and a non-coding RNA gene tmRNA. Moreover, the *Cardinium* cSfur genome harbors 31 proteins containing signal peptide, 184 proteins with transmembrane helices and 52 insertion sequences (IS). The 795 protein coding genes were classified into 726 homologous gene clusters, of which 508 (68.97%) were assigned to NCBI clusters of orthologous genes (COG) functional categories (Additional file 3). Out of the four major catogories, the “information storage and processing” accounts for 40.35%, the “metabolism” accounts for 23.23%, the “cellular processess and signaling” accounts for 20.67%, while the “poorly characterized” accounts for 15.75%.

**Table 1:** General features of genomes of the *Cardinium* strains

Bacterial Genomes	*Cardinium* cSfur	*Cardinium* cBtQ1	*Cardinium* cEper1
Host	*Sogatella furcifera*	*Bemisia tabaci*	*Encarsia pergandiella*
Contigs	1	11	1
Genome size (bp)	1,103,593	1,012,588	887,130
GC%	39.2	36.1	36.6
CDS	795	709	841
Average gene length (bp)	1052	1033	911
Coding density (%)	75.8	72.3	86.4
tRNA	35	35	37
rRNA	3	3	3

### Taxon status of *Cardinium* cSfur

**Fig. 1:**
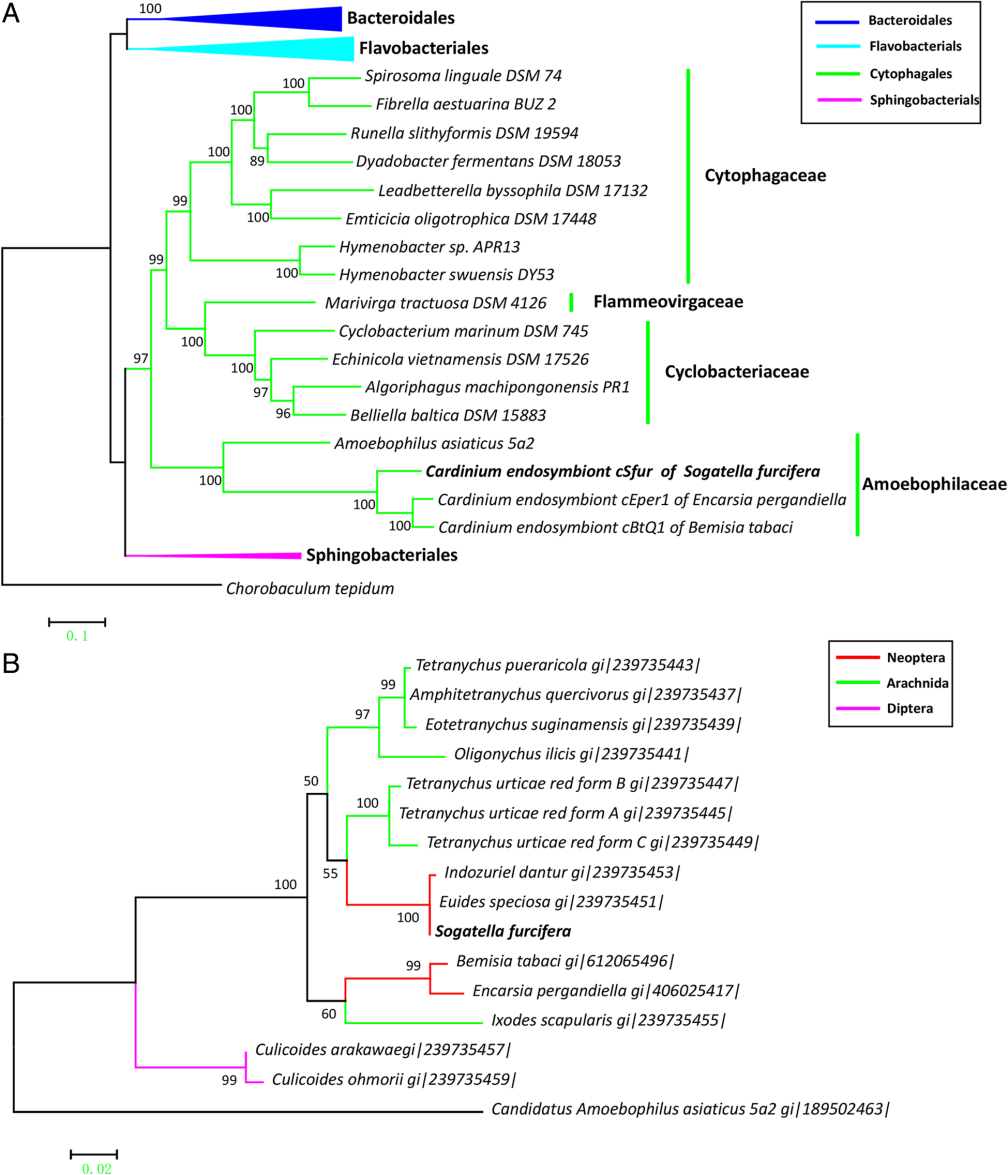
Phylogenomic tree for *Cardinium*. **a** Phylogenetic tree of 47 species. Phylogenetic tree reconstruction was done with a concatenated alignment of 16 orthologous single copy genes related to replication/recombination/repair, translation/ribosomal structure/biogenesis and post-translational modification/protein turnover. The *Cardinium* endosymbiont cSfur of *Sogatella furcifera* is displayed in bold. Species of different orders are displayed in different colors. *Chorobaculum tepidum* was used as an outgroup. Triangles represent collapsed branches of the same order. **b** Phylogenetic tree for *Cardinium* from different arthropod species based on gyrB gene. Phylogenetic relationship among different *Cardinium* supergroups are shown. The taxon names are the *Cardinium* endosymbionts’ hosts names and the geneinfo (gi) numbers of the corresponding gyrB genes. *Cardinium* cSfur falls in the clade of *Cardinium* endosymbionts of the planthopper, belonging to the group A. *Amoebophilus asiaticus* 5a2 was used as an outgroup. Bootstrap values over 50 are displayed

The phylogenetic maximum likelihood tree was reconstructed with 16 orthologous single copy genes identified from the 47 genomes. In a previous study, the Amoebophilaceae family was proposed to define the clade comprised of *Cardinium* cEper1, *Cardinium* cBtQ1 and the obligate amoeba symbiont *Amoebophilus asiaticus* [59]. As expected, the phylogenetic analysis showed that the three *Cardiniums* (cSfur, cEper1 and cBtQ1) were clustered together, and with *A. asiaticus*, distant from the other family members of Cyclobacteriaceae, Cytophagaceae, and Flammeovirgaceae in the order Cytophagales (Fig. 1a). A phylogenetic analysis with the gyrB gene revealed that *Cardinium* cSfur is closely clustered with other *Cardinium* strains from Delphacidae (*Euides speciosa* and *Indozuiel dantur*), and diversified from the clade comprising *Cardinium* cEper1 and *Cardinium* cBtQ1 (Fig. 1b).

### Genome comparison analyses of *Cardinium* cSfur

**Fig. 2:**
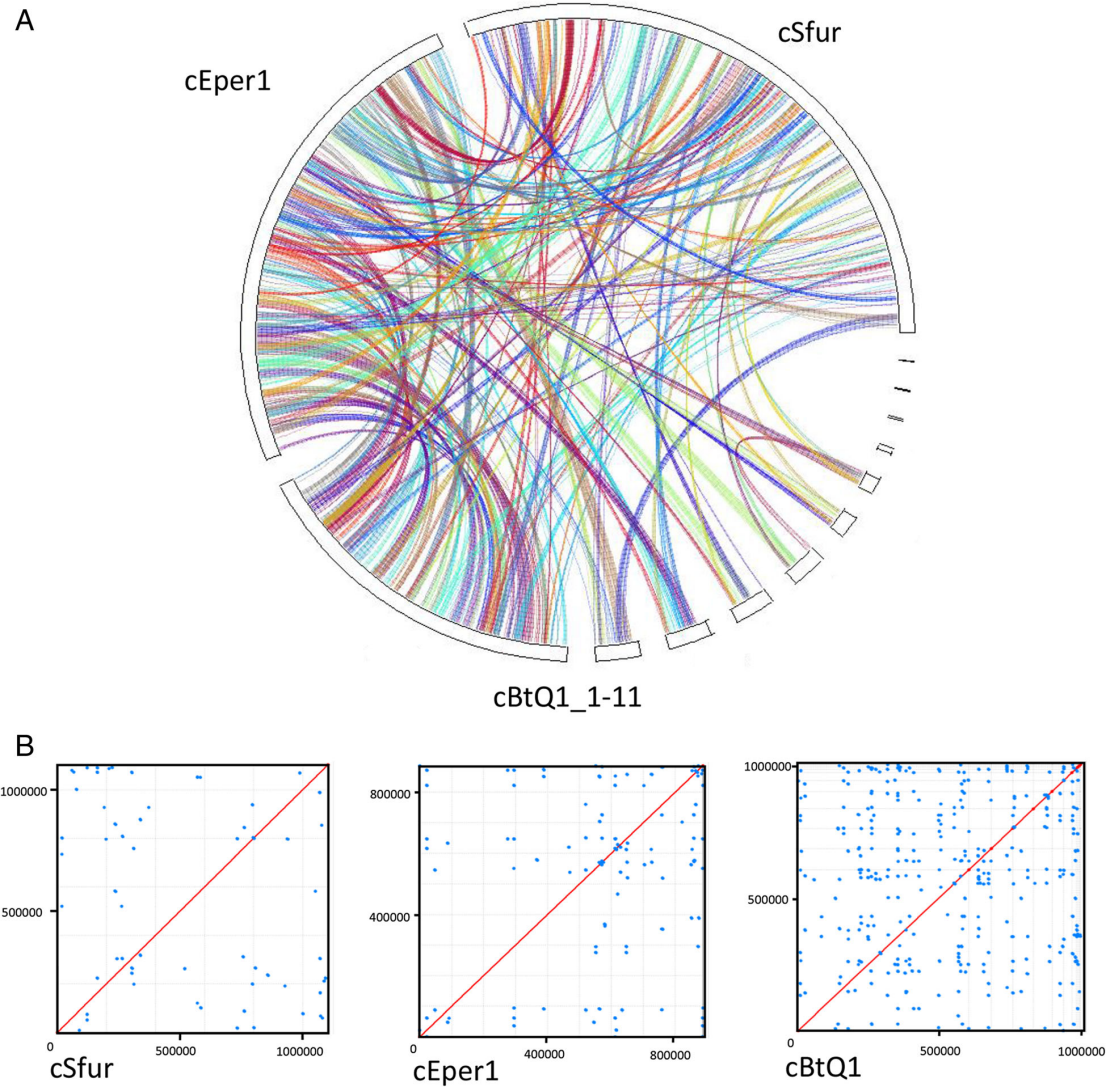
Synteny and redundancy of the three *Cardinium* genomes. **a** Synteny of the three *Cardinium* genomes at the protein level. **b** Mummer plot showing the repeats of the genomes with a minimum length of 500 bp

**Fig. 3:**
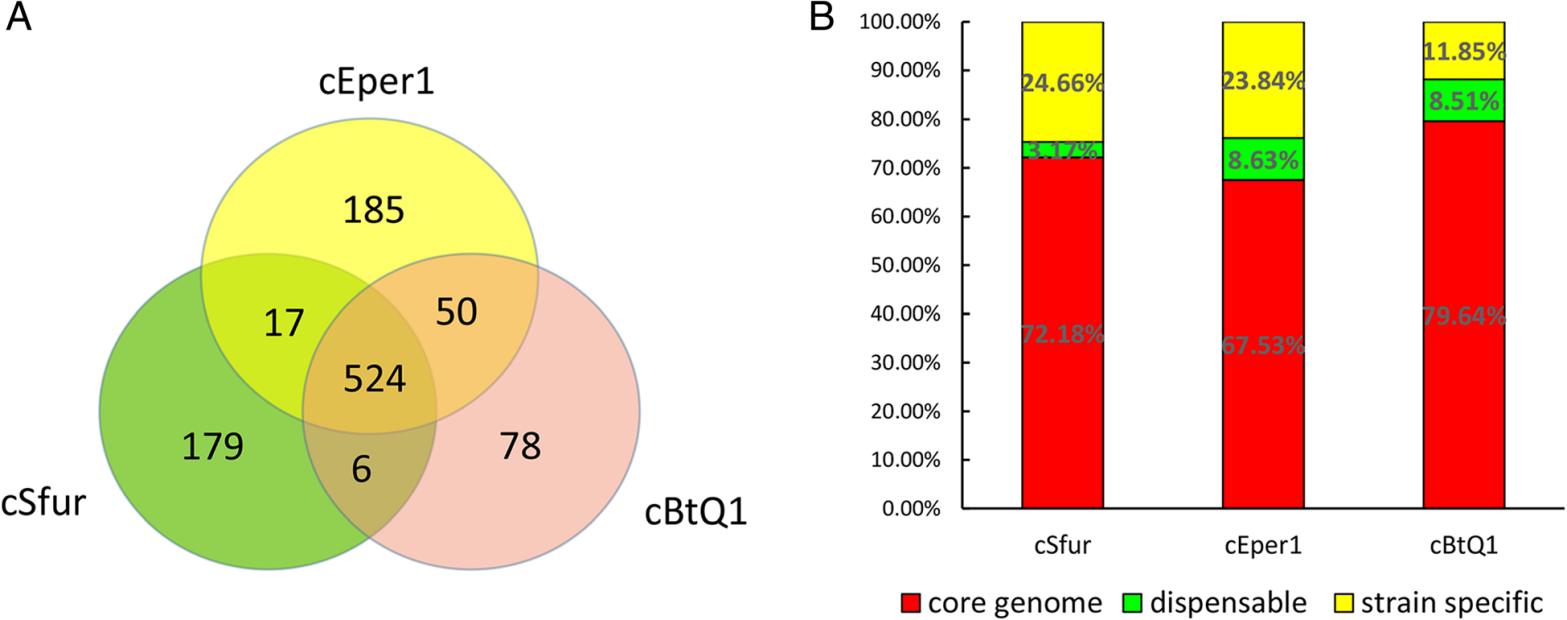
**a** Euler diagram of orthologous clusters. Euler diagram representing the core genome, the strain-specific orthologous clusters and the orthologous clusters shared only bye two *Cardinium* genomes. **b** Percentages of core, dispensable, and specific genes in three *Cardinium* genomes. The box of the core genome set is displayed in red; the genes with the highest (524) numerical values in all three genomes belong to the core genome, especially cBtQ1, which accounts for approximately 79.64%. The box of the strain-specific set is shown in yellow, the *Cardinium* cSfur has the most strain-specific genes, accounting for 24.66%

The phylogenetic analysis showed that the three *Cardinium* strains have the closest relationship (Fig. 1a). The average nucleotide identity (ANI) between the *Cardinium* cBtQ1 and cEper1 strains is 90.77%, much higher than that between both (the *Cardinium* cBtQ1 and cEper1 strains), and *Cardinium* cSfur (78.44% and 78.59% respectively). However, a significant number of homologous proteins were identified among the three *Cardiniums* at protein levels across the whole genomes (Fig. 2a). The redundancy level (Fig. 2b) of *Cardinium* cSfur (5.95%) is similar to that of *Cardinium* cEper1 (5.74%) but less than that of *Cardinium* cBtQ1 (17.7%). *Cardinium* cSfur has a similar genome size to cBtQ1, containing the same number of protein coding genes with both cBtQ1 and cEper1 (Table 1). The GC content of *Cardinium* cSfur (39.2%) is higher than that of cBtQ1 (36.1%) or cEper1 (36.6%). The coding density of the cSfur is approximately 3.5% higher than cBtQ1 and approximately 10% lower than cEper1. The core genome shared by the three *Cardinium* strains is 524 gene clusters, accounting for the highest percentage among the three genomes (Fig. 3 and Additional file 4). The core genome includes 27 gene clusters, 10 of which are involved in glycolysis, 9 in peptidoglycan biosynthesis, and 2 in lipoate biosynthesis, while 6 others are related to interaction with the host, 3 out of which are TRP-domain containing proteins with the remaining 3 being ankyrin repeat containing proteins. *Cardinium* cSfur also shares 17 gene clusters with *Cardinium* cEper1, including those (bioF and bioB) involved in biotin synthesis, and 6 homologous gene clusters with *Cardinium* cBtQ1. The numbers of strain-specific gene clusters were 179, 185 and 78 in *Cardinium* cSfur, *Cardinium* cEper1 and the *Cardinium* cBtQ1, respectively. Of the strain-specific gene clusters, three main categories of genes accounted for the high proportion; transposases, ankyrin repeat proteins and hypothetical proteins. The strain-specific gene clusters of *Cardinium* cSfur include 41 hypothetical proteins, 21 transposases and 29 ankyrin repeat containing proteins.

Both *Cardinium* cEper1 and cBtQ1 contain plasmids. A total of 95 gene sequences of two plasmids proteins (65 in pCher of cEper1, 30 in pcBtQ1 of cBtQ1) were collected and then clustered into 77 gene clusters, of which 43 and 23 were homologous to genes in the genomes of *Cardinium* cSfur and *Amoebophilus asiaticus* 5a2 respectively (Additional file 5). Among the 65 genes in the plasmid pCher, 40 were homologous to cSfur, with protein identities ranging from 30.10–87.27%, while 21 were homologous to those of *A. asiaticus* 5a2, with identities ranging from 21.43–54.81%. Similarly, out of the 30 genes in pcBtQ1, 15 and 13 show similarity to those of the cSfur and *A. asiaticus* 5a2 genomes, with identities of 25.41–89.56% and 24.41–46.67%, respectively. This suggests that the genes of both plasmids are closer to cSfur than they are to *A. asiaticus* 5a2.

**Fig. 4:**
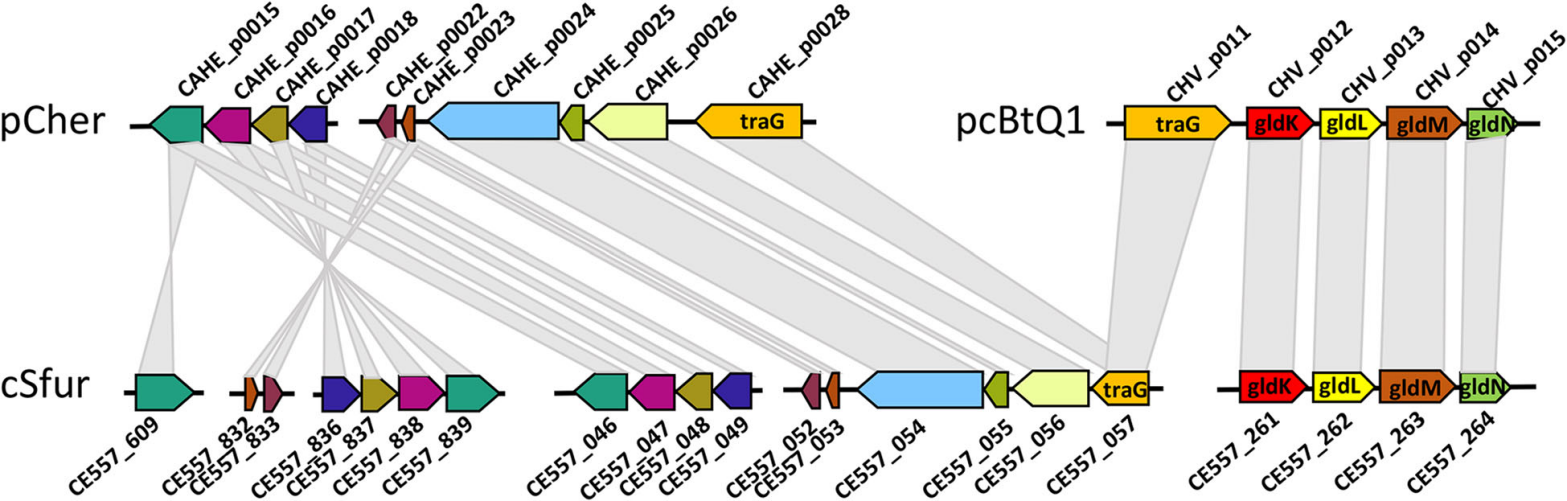
Syntenic blocks between *Cardinium* cSfur genome and *Cardinium* cEper1 and cBtQ1 plasmids. The genes marked in the same color are homologous, with pCher being the plasmid of *Caridnium* cEper1 and pcBtQ1 that of *Cardinium* cBtQ1

Three syntenic segments were identified between two plasmids and the *Cardinium* cSfur genome (Fig. 4), and 4 genes (CAHE_p0015–18) and another 6 genes (CAHE_p0022–26, CAHE_p0028) on the plasmid pCher were homologous to the genes in three (CE557_046–49, CE557_836–839 and CE557_052–57) separate regions of cSfur genome. The CE557_046–49 genes were directly homologous to the CAHE_P0015–18 genes of the plasmid, the same as CE557_836–839 but in a reverse direction and position on the cSfur genome; thus, CE557_046–49 are homologous to CE557_793–796 in reverse order, with identities of 91.45%, 89.42%, 89.31% and 95.91% respectively, while CAHE_p0022–23 are also homologous to the CE557_832–834 in the reverse direction, with identities of 90.65% and 91.14%, respectively. Five sequential genes on the plasmid cpBtQ1 (CHV_p011–15, traG and gldKLMN) showed high identities with the genes on the cSfur genome (CE557_057 and CE557_261–264). Notably, the latter four are gliding-related forming an operon. Both plasmids harbor the traG (putative conjugal recombination enzyme) gene, showing similarity to a gene (CE557_057) in the cSfur genome. Considering that most genes in the plasmids show homology with genes in the chromosomes of *Cardinium* and *Amoebophilus asiaticus* 5a2, the results imply that both plasmids may have originated in the *Cardinium* chromosome after the divergence between *Cardinium* cSfur and the last common ancestor of *Cardinium* cEper1 and cBtQ1.

**Fig. 5:**
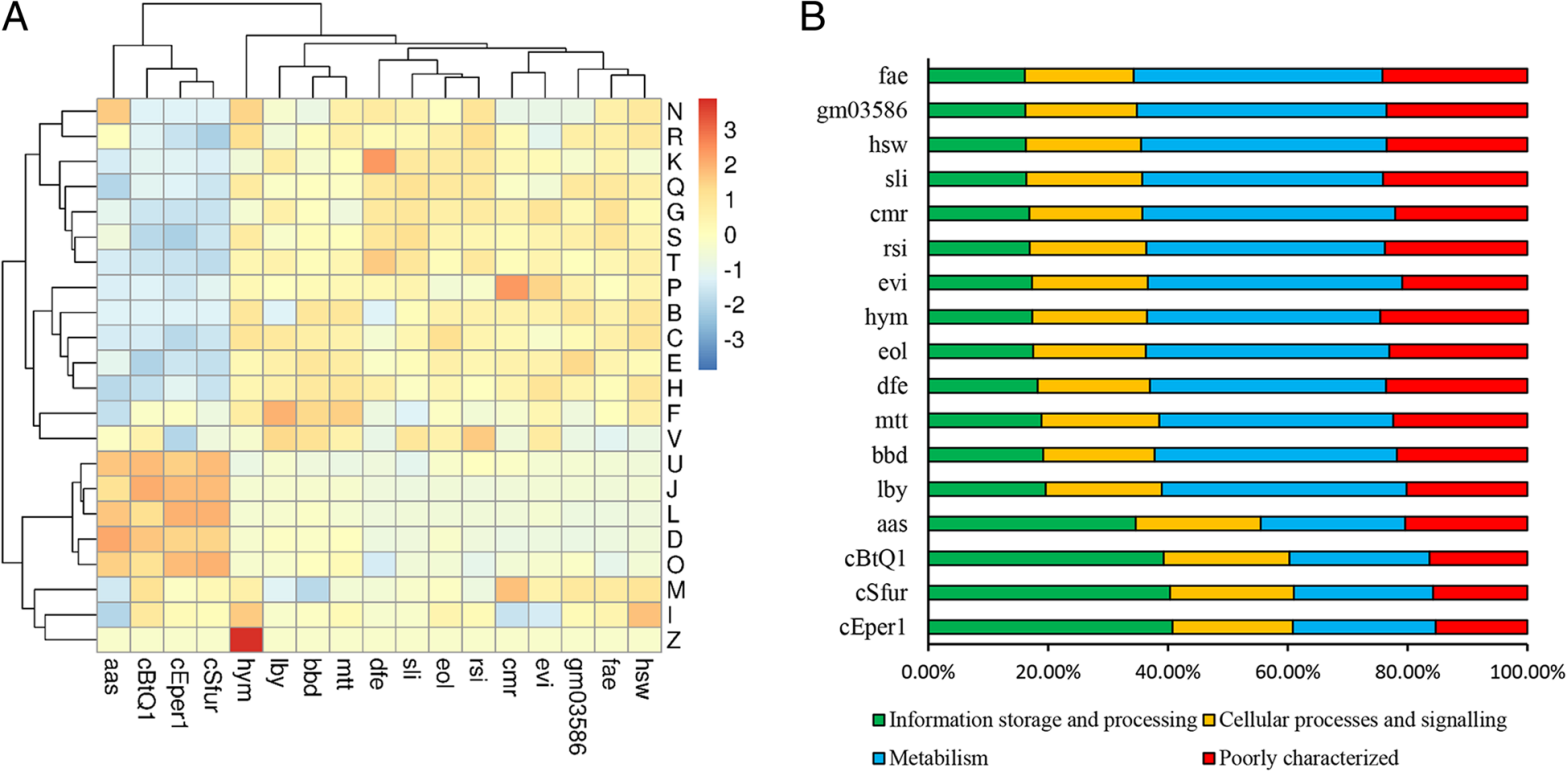
Gene distributions of 17 species in Cytophagales. **a** Percentage relative abundances of gene clusters in Cytophagales. The values are centered and scaled in the row direction. Two main COG clusters (left) are observed: highly retained categories (U, J, L, D, O, M, I, Z) and low retained categories free-living bacteria (latter 13). **b** Percentages of 4 categories in Cytophagales. Compared with the free-living bacteria, the host-dependent endosymbionts have higher percentages of genes related tothe information storage and processing (green), with lower percentages of genes involved in metabolism (blue). J: Translation, ribosomal structure and biogenesis; K: Transcription; L: Replication, recombination and repair; B: Chromatin structure and dynamics; C: Energy production and conversion; G: Carbohydrate transport and metabolism; E: Amino acid transport and metabolism; F: Nucleotide transport and metabolism; H: Coenzyme transport and metabolism; I: Lipid transport and metabolism; P: Inorganic ion transport and metabolism; Q: Secondary metabolite biosynthesis, transport and catabolism; D: Cell cycle control, cell division, chromosome partitioning; V: Defense mechanisms; T: Signal transduction mechanisms; M: Cell wall/membrane biogenesis; N: Cell motility; Z: Cytoskeleton; U: Intracellular trafficking and vesicular transport; O: Post-translational modification, protein turnover andchaperones; R: General function prediction only; S: Function unknown. Aas: *Amoebophilus asiaticus* 5a2; cBtQ1: *Cardinium* cBtQ1; cEper1: *Cardinium* cEper1; cSfur: *Cardinium* cSfur; lby: *Leadbetterella byssophila* DSM 17132; bbd: *Belliella baltica* DSM 15883; mtt: *Marivirga tractuosa* DSM 4126; dfe: *Dyadobacter fermentans* DSM 18053; sli: *Spirosoma linguale* DSM 74; eol: *Emticicia oligotrophica* DSM 17448; rsi: *Runella slithyformis* DSM 19594; cmr: *Cyclobacterium marinum* DSM 745; evi: *Echinicola vietnamensis* DSM 17526; hsw: *Hymenobacter swuensis* DY53; fae: *Fibrella aestuarina* BUZ 2; gm03586: *Algoriphagus machipongonensis* PR1

Bacterial endosymbionts usually have a reduced genome in comparison to free-living bacteria [60]. The genome analyses of species belonging to the same order Cytophagales clearly showed that the bacterial endosymbionts have a smaller genome size and GC content compared with the free-living ones (Additional file 6). The representation of functional categories in the Cytophagales genomes was performed based on the assignment of CDSs to COGs (Additional file 7). The relative abundances of genes in each COG category among all investigated bacteria were determined and used for hierarchical clustering (Fig. 5a). A heat map revealed that the endosymbionts and the free-living bacteria were clustered separately. The numbers of gene clusters in the COG categories (N, R, K, Q, G, S. T, P, B, C, E, H, F, and V) of endosymbionts were apparently lower than those of the 13 free-living genomes. In contrast, the relative gene abundances in the COG categories (U, J, L, D, O, M, I and Z) of endosymbionts were apparently higher than the percentage of genes of the free-living bacteria. The genes in six COG categories (Q, G, P, C, E, and H) were mainly involved in metabolic processes, while the COG categories (J and L) belong to the information storage and processing categories. These COG categories could be grouped into 4 functional categories, and the percentages of genes of the different functional categories in each species are shown (Fig. 5b). The results showed that the genes involved in metabolism were significantly lower represented in endosymbionts, whereas most of genes related to the information storage and processing in endosymbionts were retained. The Wilcoxon test (Table 2) further certified that there are significant differences between the host-dependent and free-living bacteria in the percentage of the information storage and processing category, as well as in the percentages of the metabolic categories (Q, G, P, C, E, and H) exclusive of the J and I categories. The bacterial symbionts are thought to be obligately dependent on their hosts for growth and share several aspects of genome evolution with unrelated obligate symbionts, including genome reduction [56]. Although the observed difference in host-dependent bacteria might be partially explained by common evolutionary origin, our findings suggest that the genome of *Cardinium* cSfur has possibly undergone significant changes to enhance its settlement in cellular environments of *S. furcifera*.

**Table 2:** Distribution of the genes of host-dependent and free-living bacteria in COG categories

Percent Mean +/− s.d.	Code	Host-dependent	Free-living	HD vs FL
				*p*-value
Metabolism	**C**	4.37 ± 0.14	5.73 ± 0.32	**3.86E-03**
	**E**	4.75 ± 0.85	9.01 ± 0.79	**3.86E-03**
	**F**	2.38 ± 0.26	2.70 ± 0.34	0.1259
	**G**	2.37 ± 0.69	7.15 ± 1.40	**8.40E-04**
	**H**	2.04 ± 0.38	4.45 ± 0.44	**8.40E-04**
	**I**	3.82 ± 0.50	3.91 ± 0.39	1.00
	**P**	2.69 ± 0.24	5.18 ± 1.08	**8.40E-04**
	**Q**	1.25 ± 0.25	2.49 ± 0.36	**3.84E-03**
Information storage and processing	**B**	0.00 ± 0.00	0.04 ± 0.02	**0.01063**
	**J**	21.45 ± 2.66	6.71 ± 0.89	**8.40E-04**
	**K**	4.06 ± 0.18	6.03 ± 0.95	**8.40E-04**
	**L**	13.23 ± 1.43	4.67 ± 0.61	**3.86E-03**
Cellular processes and signaling	**D**	1.91 ± 0.18	0.77 ± 0.15	**8.40E-04**
	**M**	7.19 ± 0.41	7.22 ± 0.38	1.00
	**N**	0.08 ± 0.16	0.15 ± 0.09	0.1552
	**O**	5.97 ± 0.35	3.86 ± 0.44	**8.40E-04**
	**T**	1.02 ± 0.19	3.56 ± 0.53	**3.84E-03**
	**U**	2.50 ± 0.08	1.22 ± 0.15	**8.40E-04**
	**V**	2.02 ± 0.32	2.24 ± 0.30	0.36440
	**Z**	0.00 ± 0.00	0.01 ± 0.03	**3.36E-03**
Poorly characterized	**R**	12.29 ± 1.28	14.48 ± 0.88	**8.40E-04**
	**S**	4.63 ± 1.21	8.42 ± 0.79	**3.86E-03**

*p*-values < 0.05 are shown in bold to indicate significant differences between host-dependent and free-living bacteria (Wilcoxon test)

### Biosynthetic and transport capabilities in *Cardinium* cSfur

According to the KEGG classification pathways, *Cardinium* cSfur presents low biosynthetic capabilities. For 174 KEGG metabolism pathways, the number of complete, incomplete and non-existent metabolism pathways in *Cardinium* cSfur were 4, 39 and 131, respectively (Additional file 8). In contrast, the number of complete, incomplete and non-existent metabolism pathways in its free-living relatives *Emticicia oligotrophica* DSM 17448 and *Leadbetterlla byssophila* DSM 17132 were 29, 67, 78 and 28, 62, 84, respectively (http://www.kegg.jp/kegg/genome.html; Additional file 8). The virtually complete pathways identified in *Cardinium* cSfur only include biotin metabolism, lipoic acid metabolism, peptidoglycan biosynthesis and glycolysis.

Biotin is a coenzyme belonging to the vitamin B class and is necessary for cell growth, and the production of fatty acids and amino acids [61]. This B-vitamin is thus an indispensable nutritional factor for insect growth and metamorphosis [62]. Biotin cannot be synthesized by eukaryotes, including insects, but a complete pathway of biotin synthesis (bioA, bioD, bioC, bioH, bioF, bioB) was identified in *Cardinium* cSfur and *Wolbachia* wSfur (GenBank accession number: MH210682-MH210687), both of which co-existed in *S. furcifera*. Most biotin synthesis genes in the *Cardinium* cSfur and *Wolbachia* wSfur showed higher identity with their relatives (*Cardinium* cEper1 or cBtQ1 and *Wolbachia*. sp), respectively (Additional file 2). Interestingly, the HGT analysis revealed that the presence of a complete biotin operon in *Cardinium* is likely to be an event of acquisition of foreign genes from an Alpha-proteobacteria species, perhaps the co-inhabiting *Wolbachia*.

Lipoate is a highly conserved organosulfur co-factor that is required for the function of several key enzyme complexes in intermediate metabolism and an important antioxidant molecule [63]. Like in the two other *Cardinium* genomes (cEper1 and cBtQ1), two key enzymes (LipA and LipB) of the lipoate biosynthesis pathway were found in the *Cardinium* cSfur genome, which suggests an ability to synthesize lipoate. The *Cardinium* cSfur genome also includes PGN synthetic enzymes (murA-F, mraY, murG, mrcA and ftsI) and several lipopolysaccharide (LPS) synthetic enzymes (lpxA-D, lpxH, lpxK, lpxL). However, *Cardinium* cSfur lacks the lpxM and KdtA genes responsible for encoding acyltransferase and glucosyltransferase, respectively, and thus it cannot synthesize LPS and may not induce a host immune response [23].

Many incomplete biosynthetic pathways were also identified in the *Cardinium* cSfur genome. For example, the *Cardinium* cSfur genome contains all genes required for fatty acid biosynthesis except the key fatty acid synthase gene responsible for synthesizing the acetyl-acyl-carrier protein. The *Cardinium* cSfur genome also harbors many genes involved in the biosynthesis of purine and pyrimidine but lacks the genes responsible for the initial steps of these processes. Being an obligate endosymbiont, *Cardinium* cSfur may be supplemented with intermediate metabolites or enzymes by the host to facilitate the synthesis of fatty acids and nucleotides.

Eighty (80) transport proteins (Additional file 9) were identified with the BLASTP against TCDB (transporter classification database), accounting for 10.06% of all the 795 protein-coding genes. While the proportion of the transport proteins number in *Cardinium* cEper1 and *Amoebophilus asiaticus* 5a2 are 7.13% (60/841) and 5.27% (82/1557), respectively. These transporters were classified into 44 families, with the ATP-binding cassette (ABC) superfamily containing 12 genes, making it possible for the endosymbionts to uptake nutrients from the host. Moreover, *Cardinium* can uptake ATP from the host via the ATP: ADP antiporter (CE557_457 and CE557_682), and dicarboxylates via C4-dicarboxylate uptake proteins (CE557_216, CE557_463 and CE557_464). The existence of the transporters may compensate for the low biosynthetic capabilities of *Cardinium* cSfur genome.

### Horizontal gene transfer in *Cardinium* cSfur

**Fig. 6:**
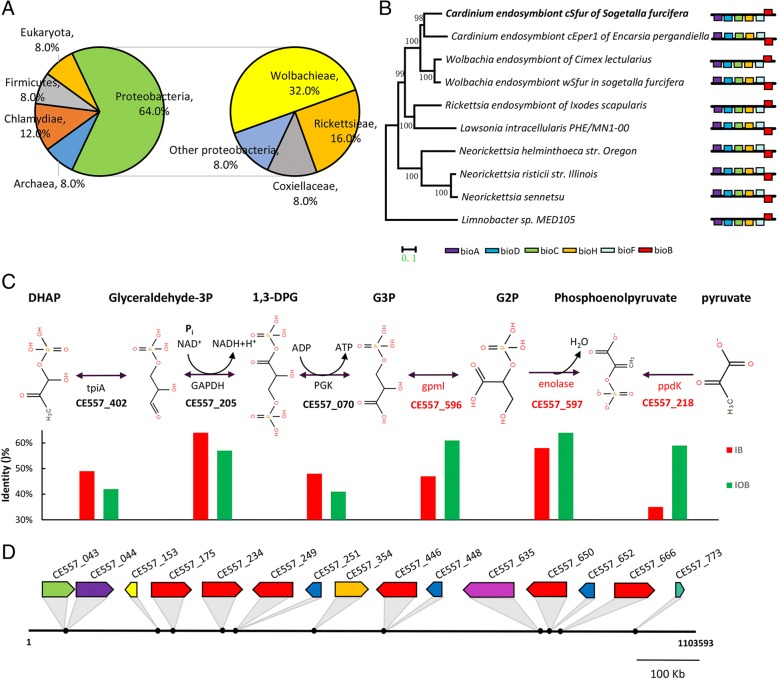
HGT in the *Cardinium* cSfur genome. **a** Percentages of nearest neighbors of the 25 genes encoding non-transposases acquired by HGT in *Cardinium* cSfur genome. **b** Phylogenetic tree of the genes related to biotin biosynthesis. Ten species harboring all the 6 genes were chosen, the 6 encoding proteins of each genome were concatenated in order and aligned with MAFFT, and then phylogenetic trees were reconstructed using the software MEGA6. **c** Genes related to glycolysis in the *Cardinium* cSfur genome and the highest identity between each gene and its homologous ones inside (red) or outside (green) of the Bacteroides phylum (IB or IOB). **d** The 15 genes encoding transposases aquired by the event of HGT in the *Caridnium* cSfur genome, the genes marked in the same color are homologous

Horizontal gene transfer (HGT) refers to the acquisition of foreign genes by organisms. HGT is a crucial mechanism contributing to bacterial adaptability and diversity [64]. Evidence of HGT discovery in the completely sequenced genomes was revealed by a deviant composition of acquired genetic elements (GC content, codon usage), a high similarity of genes to distantly related species, the variation of gene content between closely related strains, and incongruent phylogenetic trees [65]. Based on the GC content and higher protein similarities to distantly related species, 40 genes (5.03% of the 795 protein coding genes) were identified as HGT genes in *Cardinium* cSfur (Additional file 2) acquired from the organisms outside of Bacteroides. In addition, 15 HGT genes showed similarities to transposase; the other 25 encoded non-transposases, out of which 16 (64%) showed the highest similarities with proteins existing in the Proteobacteria phylum, of which 8 (32%) may be transferred from *Wolbachia*, and 4 (16%) from Rickettsia respectively (Fig. 6a). The occurrence of HGT events were inferred from the reconstructed phylogenetic tree, with 19 genes acquired before the divergence between *Cardinium* cSfur and other *Cardinium* genera, and the other 21 acquired after the divergence (Additional file 10).

As discussed above, *Cardinium* cSfur presents a complete biotin biosynthetic pathway comprising 6 genes (CE557_856–861). The separate phylogenetic tree of these 6 genes (Additional file 2, A-F) reconstructed with their top 50 BLASTP hits suggests that all of the 6 genes of *Cardinium* genera are very close to *Wolbachia*. In addition to the *Wolbachia* genera, most of the top 50 hits of these 6 genes are from Proteobacteria species including Rickettsiales. Thus, it was perceived that the 6 genes related to biotin synthesis widely exist in Proteobacteria. Therefore, HGT analysis revealed that the biotin operon in *Cardinium*, which is an ancient event of HGT from Alpha-proteobacteria, might be transferred from the co-inhabiting *Wolbachia*. The organization and orientation of the 6 genes were conserved among the bacteria, suggesting that the genes might be transferred in the form of a whole operon (Fig. 6b). Interestingly, the complete biotin synthesis pathway exists in *Cardinium* cEper1 also [59], whereas *Cardinium* cBtQ1 lacks the ability to synthesize biotin due to an IS insertion and a later deletion event [59]. The acquisition of the complete biotin synthesis operon suggested *Cardinium* cSfur may play role in the host nutrition.

Glycolysis is the metabolic pathway that converts glucose into pyruvate and releases free energy to form the high-energy molecules ATP and NADH (reduced nicotinamide adenine dinucleotide). The *Cardinium* cSfur genome encodes 6 genes for sequentially enzyme-catalyzed reactions in the glycolysis pathway (Fig. 6c). The three genes, triose-phosphate isomerase (CE557_402, tpiA), glyceraldehyde 3-phosphate dehydrogenase (CE557_205, GAPDH,) and phosphoglycerate kinase (CE557_070, PGK), which showed the highest protein similarities to proteins in the species of the same Bacteroides genera, were regarded as vertically transmitted genes. However, the other three genes, gpml (CE557_596, 2,3-bisphosphoglycerate-independent phosphoglycerate mutase), enolase (CE557_597) and ppdK (CE557_218, pyruvate, orthophosphate dikinase), were identified as HGT genes and acquired from other proteobacteria distantly related to the *Cardinium* genera (Additional file 2, G-I). Notably, many species of the genus Bacteroides also harbor the three genes (gpml, enolase and ppdK), but these proteins showed lower similarities with the three proteins in *Cardinium* cSfur, suggesting that *Cardinium* might have lost the three vertically transmitted genes and then horizontally re-acquired the three genes after settlement in the host (Fig. 6).

There are 15 genes acquired by the event of HGT that encode transposases in *Cardinium* cSfur genome (Additional file 2, AA-AH), of which, 4 were identified as ancient HGT genes. Based on similarities, these transposases were classified into 8 groups (Fig. 6d), of which CE557_043 may be acquired by the event of horizontal gene transfer from Chloroflexi species, and the other 7 groups may be laterally transferred from Proteobacteria. The CE557_251, CE557_448 and CE557_652 are homologous genes, as are CE557_175, CE557_234, CE557_249, CE557_446, CE557_650, and CE557_666 in respective orders. These multicopy transposons might be from independent HGT events or the subsequent duplication of these transposons after horizontal acquisition from the donor species, thus causing the genome diversity of *Cardinium*.

### Por secretion system and gliding genes in *Cardinium* cSfur

The Por secretion system (PorSS), a novel protein secretion system, has been found in most genera and species of the phylum Bacteroidetes [66]. For instance, the PorSS of *Flavobacterium johnsoniae* secretes chitinases required for digesting chitin [66]. The core PorSS genes (gldK, gldL, gldM, gldN, sprA, sprE, and sprT) were screened in the *Cardinium* cSfur genome, and 4 genes (gldK, gldL, gldM and gldN) related to the PorSS system were identified. The four genes formed an operon with the space regions, 63, 81 and 20 nucleotides in respective orders. Orthologs of the 4 PorSS genes were also identified from the plasmid of *Cardinium c*BtQ1*.* Our data thus suggests that PorSS is required for the secretion of the cell surface and extracellular proteins in both *Cardinium* cBtQ1 and *Cardinium* cSfur.

**Fig. 7:**
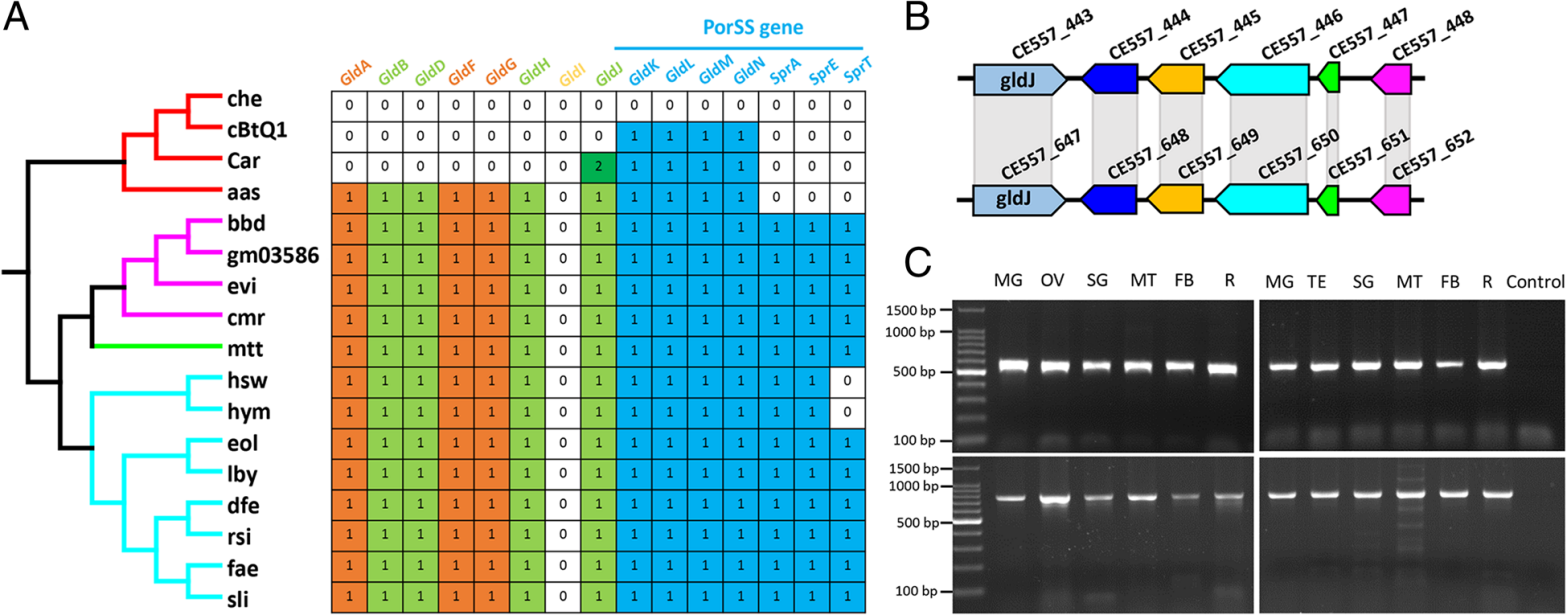
Gliding genes profile, PorSS system and PCR confirmation of *Cardinium* cSfur existence. **a** Distribution of gld and spr orthologs in the Cytophagias. The genes gldKLMN and sprAET also encode the PorSS system. **b** Duplication of gldJ and the following 5 genes in the *Cardinium* cSfur genome. **c** PCR confirmation of *Cardinium* cSfur existence in different tissues of *S. furcifera*. PCR of a fragment of 16S rDNA (766 bp) and gyrB (575 bp) gene of different tissues of female and male adults. MG: malpighian tubule, OV/TE: ovary/testis, SG: salivary gland, MT: midgut, FB: fat body, R: rest of body, control: negative control without DNA template

The PorSS genes are also an integral part of the gliding motility machinery in the bacteria of the phylum Bacteroidetes, in that PorSS is necessary for assembly of the motility apparatus [66]. The core set of genes required for bacteroidete gliding motility includes 15 (gldA, gldB, gldD, gldF, gldG, gldH, gldI, gldJ, gldK, gldL, gldM, gldN, sprA, sprE and sprT) genes. In comparison with other free-living bacteria of the same order Cytophagales, species in the *Cardinium* genera lost most gliding genes (Fig. 7a). However, two copies of gldJ genes (CE557_443 and CE557_647) were identified in the *Cardinium* cSfur genome. These two genes might be derived from chromosomal segmental duplication, as the case is with the 5 genes preceded by the two gldJ (CE557_443 and CE557_647) genes. The 5 genes (CE557_444–448) preceded by the gldJ (CE557_443) gene had a 100% identity with the 5 genes (CE557_648–652) preceded by the gldJ (CE557_647) gene in respective orders (Fig. 7b). In addition to being a component of the gliding motility machinery, evidence in support of other functions of gldJ in vivo was reported in *Flavobacterium johnsoniae*, where a localization of gldJ by immunofluorescence microscopy and transmission electron microscopy revealed that most of the gldJ were not exposed on the cell surface [67]. Thus, the duplication of gldJ and the following 5 genes in the *Cardinium* cSfur genome may have little or no direct relationship with the gliding motility.

The PCR detection of the 16S rRNA and gyrB gene of *Cardinium* cSfur were positive in the malpighian tubule, ovary/testis, salivary gland, midgut, fat body and the rest of the body of the *S. furcifera* host (Fig. 7c), suggesting that *Cardinium* cSfur has a mobile capability. Similar phenotypes were observed in *Cardinium* cBtQ1 [9], different from the *Cardinium* cEper1 which is restricted to the ovary of its host [59]. Also, the mobility of both *Cardinium* cBtQ1 and *Cardinium* cSfur might also be due to other mechanisms reliant on the presence of rtxBDE/tolC and mreB genes. TolC, an outer membrane protein, possesses the ability to efflux a transmembrane transpoter [68], while mreB, an actin-like protein, was identified to be essential for bacterial motility [69]. The genes tolC (CE557_408) and mreB (CE557_360) were also found in the *Cardinium* cSfur genome, with approximately 52% and 95% identities between them and those of cBtQ1 respectively. The existence of the genes tolC and mreB may substitute for the function of other gliding genes absent in the *Cardinium* cSfur genome.

### *Cardinium* cSfur as probable secondary endosymbionts (S-endosymbionts)

**Fig. 8:**
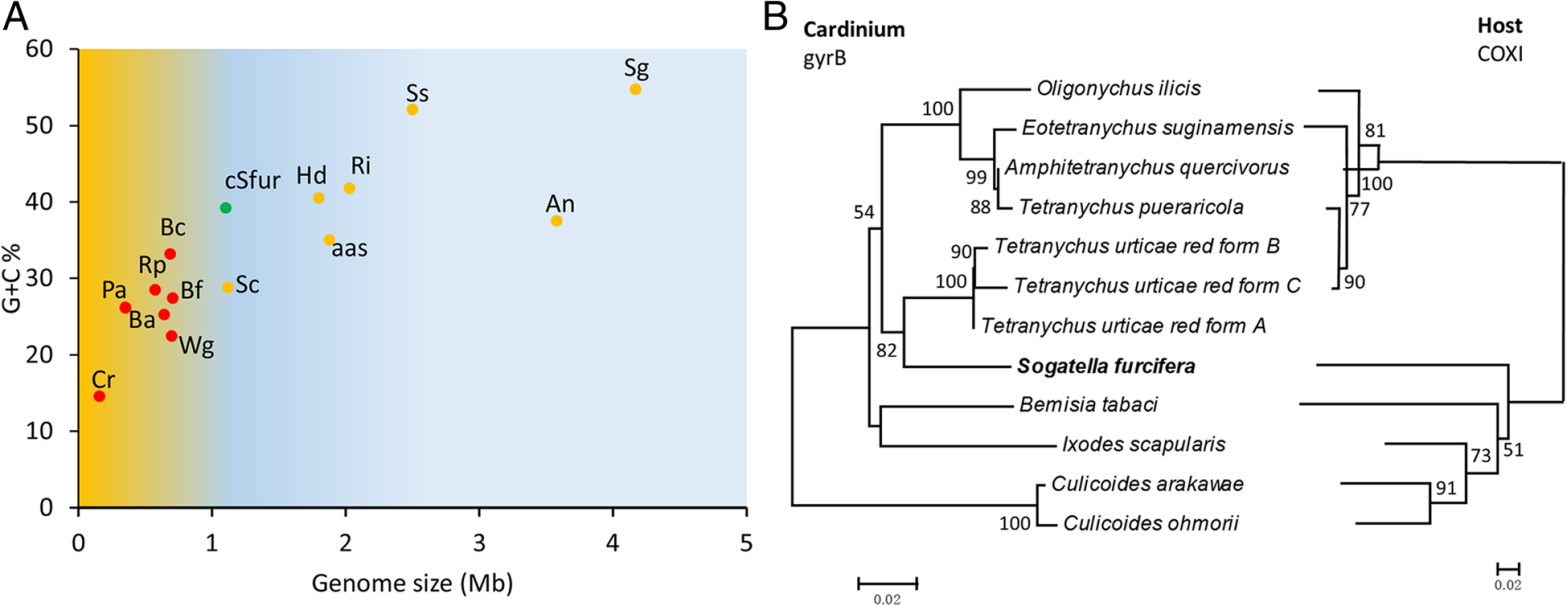
Genome size comparison and evaluation of evolutionary relationships between *Cardinium* and their hosts. **a** Comparison of genome sizes and GC content of selected endosymbiotic bacteria. P-endosymbionts are marked in red, S-endosymbionts in orange, and *Cardinium* cSfur is specially marked in green. Cr, *Carsonella ruddii* DC; Pa, *Portiera aleyrodidarum* BT-B-HRs; Rp, *Riesia pediculicola*; Ba, *Buchnera aphidicola* str*.* Sg; Bc, *Baumannia cicadellinicola*; Bf, *Blochmannia floridanus*; Wg, *Wigglesworthia glossinidia*; Sc, *Spiroplasma chrysopicola*; Hd, *Hamiltonella defensa* 5AT; aas, *Amoebophilus asiaticus* 5a2; Ri, *Regiella insecticola* R5.15; Ss, *Serratia symbiotica* str. Tucson; An, *Arsenophonus nasoniae*; Sg, *Sodalis glossinidius*. **b** Comparisons of the evolutionary relationships between *Cardinium* strains and their different insect hosts. The host tree is based on the mitochondria gene COXI sequence, while the endosymbiont *Cardinium* tree is based on gyrB

The genome sequences of symbiotic bacteria revealed their smaller genomes compared with their free-living relatives, and the insect endosymbionts’ genomes tend to be reductive [70], especially the P-endosymbionts [71]. Published data of several P- and S-endosymbionts, including genome size and GC content, were collected (Additional file 6). A scatter plot (Fig. 8a) emphasizes a positive correlation between genome size and GC content, and shows that the P-endosymbionts (marked in red) are clustered with a lower GC content and smaller genome size compared with the S-endosymbionts (orange dots), which span a wider spectrum of genome size. *Cardinium* cSfur (marked in green) can be classified as an S-endosymbiont since its GC content is relatively higher than that of the P-endosymbionts, and it has a larger genome size. The *Cardinium* cSfur genome size is almost the same as that of *Spiroplasma chrysopicola* [72], which has been described as an S-endosymbiont [73]. The plot (Fig. 8a) thus suggests that *Cardinium* cSfur may be an S-endosymbiont undergoing genome reduction.

P-endosymbionts are described to show co-evolution with their hosts while the S-endosymbionts do not [3]. To ascertain if *Cardinium* belongs to the P- or S-category of endosymbiont, the gyrB genes of 12 *Cardinium* strains were used to reconstruct a phylogenetic tree of the *Cardinium* genera. In a like manner, the mitochondrial gene COXI (cytochrome C oxidase subunit I) of 10 *Cardinium* hosts including *S. furcifera* were used to reconstruct a phylogenetic tree of the hosts (Fig. 8b). The results indicate that the evolutionary relationship of the *Cardinium* species differ from that of their hosts, thus implying that *Cardinium* cSfur may be an S-endosymbiont.

## Discussion

We present the first report of the complete genome sequence of a novel *Cardinium* cSfur strain in *S. furcifera*. As far as we know, information has only been provided on the complete genomes of *Cardinium* cBtQ1 (endosymbiont of whiteflies) and *Cardinium* cEper1 (endosymbiont of parasitoids) [9, 59]. Recently, our laboratory obtained the genome sequence of *S. furcifera* by the WGS approach using genomic DNA isolated from the insect [25]. Notably, earlier studies had reported the infection of the natural population of *S. furcifera* with *Cardinium* strains [24]. In line with this assertion, our group assembled the *Cardinium* genome from raw reads of the whole genome sequence of *S. furcifera*.

.

Principally, the genome sequence of *Cardinium c*Sfur reveals a high reduction, both in genome size and metabolic pathways. The obligate bacterial endosymbiont has lost many genes that are commonly found in closely related bacteria [60]. Most intracellular bacteria have extremely small genomes compared with their free-living counterparts [71]. Primary endosymbiotic insects and quite a number of obligate pathogens such as *Mycoplasma*, *Ureaplasma*, *Rickettsia* and *Chlamydia* also possess relatively small genome sizes [74, 75]. Our analysis of the 17 members of the order Cytophagales to which *Cardinium* belongs showed that the 4 bacterial endosymbionts of the order had relatively lower genome sizes when compared with their 13 free-living counterparts. We thus inferred that genomic size reduction is a peculiar trait of most bacterial endosymbionts. Our findings are consistent with those of [70], who compared representative genomes of some free-living bacteria and symbionts. Due to *Cardinium*’s endosymbiotic nature, we presume that some of the genes supposedly needed for its metabolic activities have been lost, since it absolutely depends on its host for metabolic support. Principally, most genes retained were those implicated in information storage and processing. The observable genome size reduction thus suggests that the *Cardinium* genome has undergone significant changes to facilitate establishment and adaptation in its host.

It is noteworthy that despite its metabolically restricted genome, *Cardinium* encodes the complete biosynthesis pathways for biotin and lipoate, which play potential roles in host nutrition. Other prominent examples of the predicted genes implicated in horizontal gene transfer are three genes related to the glycolytic pathway. Correspondingly, *Cardinium* cSfur gets a supply of some metabolites from the host to facilitate its survival, hence confirming the mutually beneficial relationship. Essentially, *Cardinium* encodes a set of proteins with the potential to interfere with eukaryotic cell cycle regulation such as the ankyrin repeat containing proteins (ANK) and tetratricopeptide repeat containing proteins (TRP). Additionally, 80 transport proteins were identified in the genome of *Cardinium* cSfur. The proteins are perceived to act as the hub between the endosymbiont and its host, thereby compensating for its reduced metabolic capabilities. A similar study [59] reported that *Cardinium* cEper1 encodes 60 transport proteins that help in its metabolic activities.

Our result suggests that *Cardinium* cSfur is a probable member of the Secondary endosymbiotic bacterial group (Fig. 8). Our presumption is based on their possession of larger genome size, higher GC content, limited metabolic capability and a non co-evolution with the host. A large genome size and higher GC content have been reported as defining features of S- endosymbionts [76]. The phylogentic tree (Fig. 8b) showed that the *Cardinium* strains had no co-evolution with their host which further confirms their secondary endosymbiotic nature. The secondary endosymbiotic nature of *Cardinium* makes it less involved in the metabolic pathway. Nevertheless, its host supplies the metabolites needed for energy generation and other vital processes.

## Conclusions

The genomic analysis of this novel bacterial endosymbiont has provided a further understanding of its symbiotic relationship with its *Sogetella furcifera* host. Our findings revealed that the *Cardinium* cSfur genome changed significantly to adapt to the symbiotic relationship with its host. Remarkably, a horizontal gene transfer event was also observed between *Cardinium* cSfur and its donor organisms- *Wolbachia* and *Rickettsia*. The genomic information on *Cardinium* cSfur will be helpful in understanding how it has undergone changes to facilitate its settlement in *S. furcifera*. It will also enhance the development of an endosymbiont-based and eco-friendly control mechanism for the perpetually devastating agricultural pest.

## Additional files

Additional file 1: Microbial Genome Database organism codes and locus tags for the genes used in the phylogenomic tree. (XLSX 105 kb)

Additional file 2: Phylogenetic trees of the genes acquired by the event of HGT in the Cardinium cSfur genome. (PDF 970 kb)

Additional file 3: Clusters of orthologous genes (COG) functional classification of the Cardinium cSfur genome (gene homologous cluster number). (PDF 276 kb)

Additional file 4: Gene clusters among three Cardinium genomes. (XLSX 56 kb)

Additional file 5: Genes in the two Cardinium plasmids (pcBtQ1 and pcher) and their homologous genes in the genomes of Cardinium cSfur and Amoebophilus asiaticus 5a2. (XLSX 11 kb)

Additional file 6: General features of genomes of 17 cytophagales (A) and P- and S- endosymbiont genome size and GC content (B). (XLSX 12 kb)

Additional file 7: COG category cluster numbers in 17 bacteroides species. (XLSX 10 kb)

Additional file 8: Pathway statistics of Cardinium cSf. (PDF 85 kb)

Additional file 9: Transport proteins in the Cardinium cSfur genome. (XLSX 15 kb)

Additional file 10: Genes acquired by the event of HGT in the Cardinium cSfur genome. (XLSX 14 kb)

## Abbreviations

CDS: coding DNA sequence
CFB: Cytophaga-Flavobacterium-Bacteroides
CI: Cytoplasmic incompatibility
COG: Clusters of orthologous genes
COXI: Cytochrome C oxidase subunit I
HGT: Horizontal gene transfer
IS: Insertion sequence
KAAS: KEGG Automatic Annotation Server
KEGG: Kyoto Encyclopedia of Genes and Genomes
LPS: Lipopolysaccharide
NCBI: National Center for Biotechnology Information
NR: NCBI non-redundant protein database
P-endosymbiont: Primary endosymbiont
PGN: Peptidoglycan
PorSS: Por secretion system
rRNA: Ribosomal RNA
S-endosymbiont: Secondary endosymbiont
SRBSDV: Southern rice black-streaked dwarf virus
TCDB: Transport Classification Database
tmRNA: Transfer-messenger RNA
tRNA: Transfer RNA
WBPH: White-backed planthopper
