# Supplementary material for: Bacterial endosymbiont Cardinium cSfur genome sequence provides insights for understanding the symbiotic relationship in Sogatella furcifera host
Source: BMC Genomics. 2018 Sep 19;19:688. doi: 10.1186/s12864-018-5078-y (PMC6147030; doi:10.1186/s12864-018-5078-y)

Figure S1. The genes may be acquired by the event of horizontal gene transfer in *Cardinium* cSfur

A-F: Biotin synthesis genes
G-I: Glycolysis relasted genes
J-W: Other non-transposase encoding genes
AA-AH: Transposase endcoding genes

A: CE557_856 (bioA: adenosylmethionine-8-amino-7-oxononanoate transaminase)

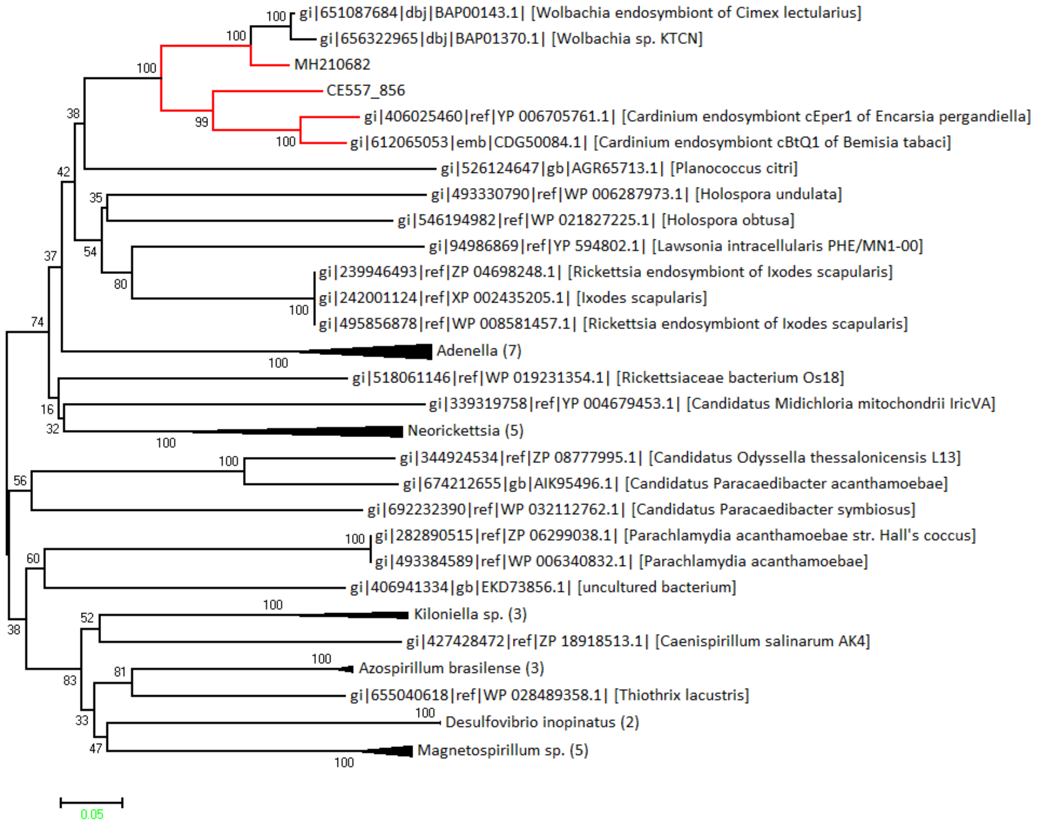

B: CE557_857 (bioD: dethiobiotin synthase)

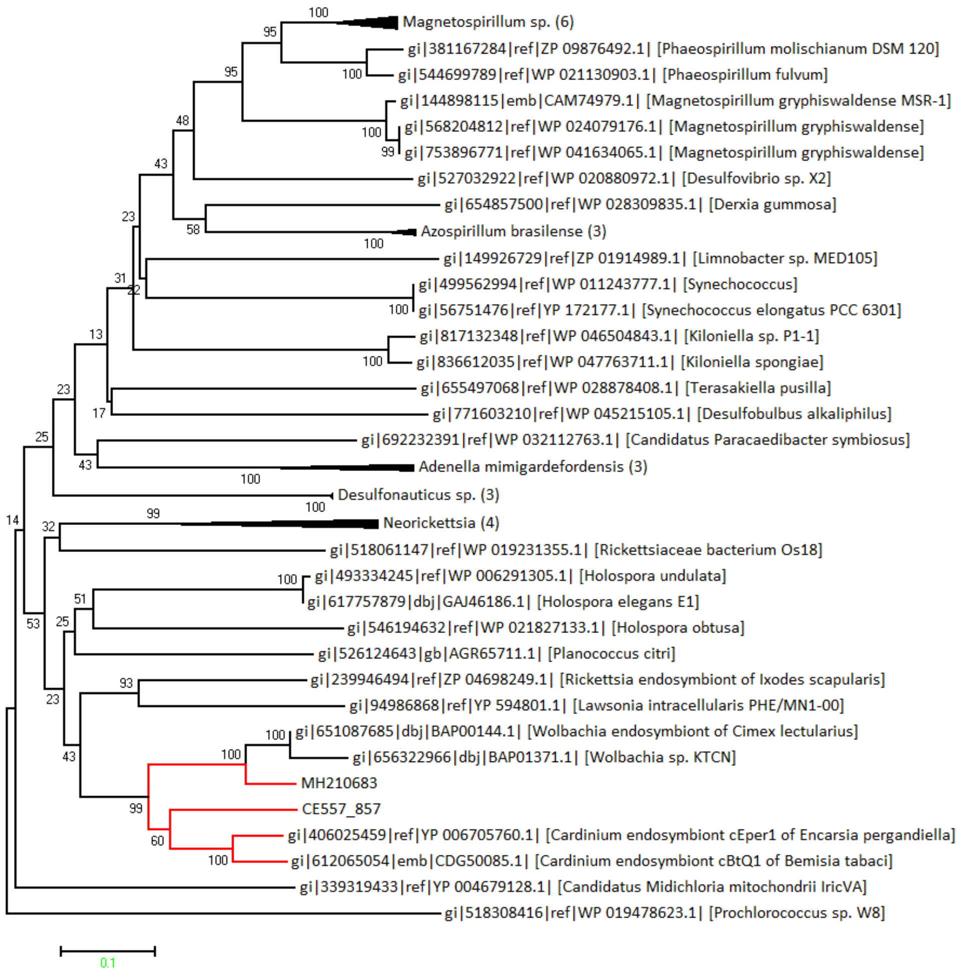

C: CE557_858 (bioC: malonyl-CoA O-methyltransferase)

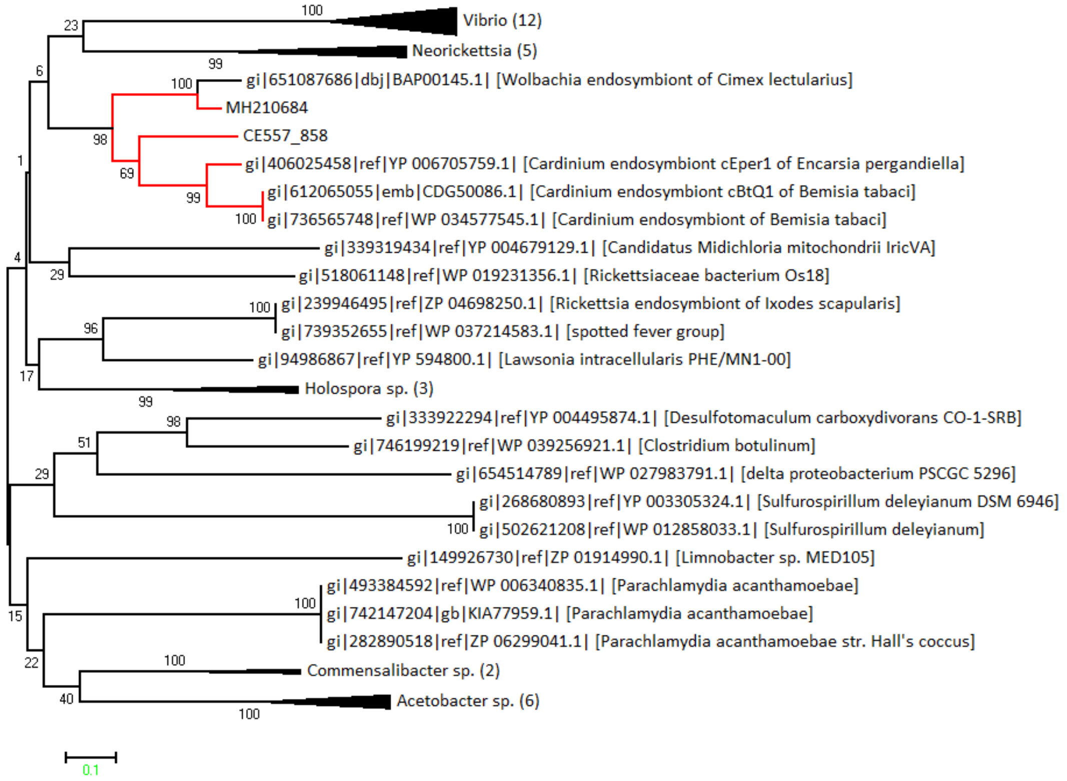

D: CE557_859 (bioH: pimeloyl-[acyl-carrier protein] methyl ester esterase)

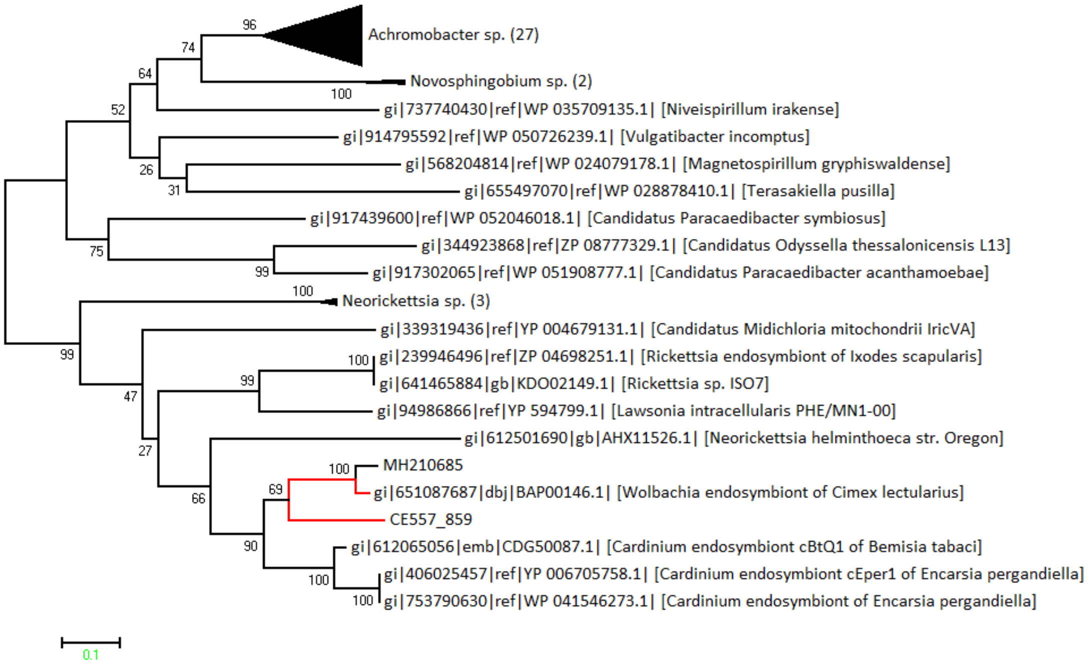

E: CE557_860 (bioF: 8-amino-7-oxononanoate synthase)

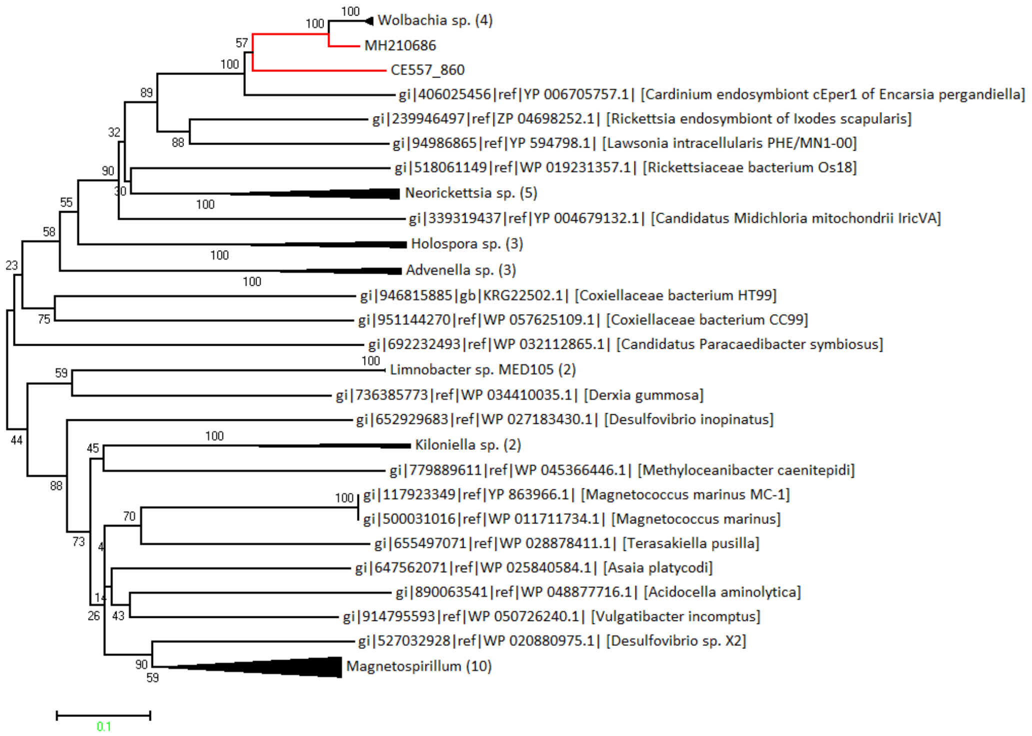

F: CE557_861 (bioB: biotin synthase)

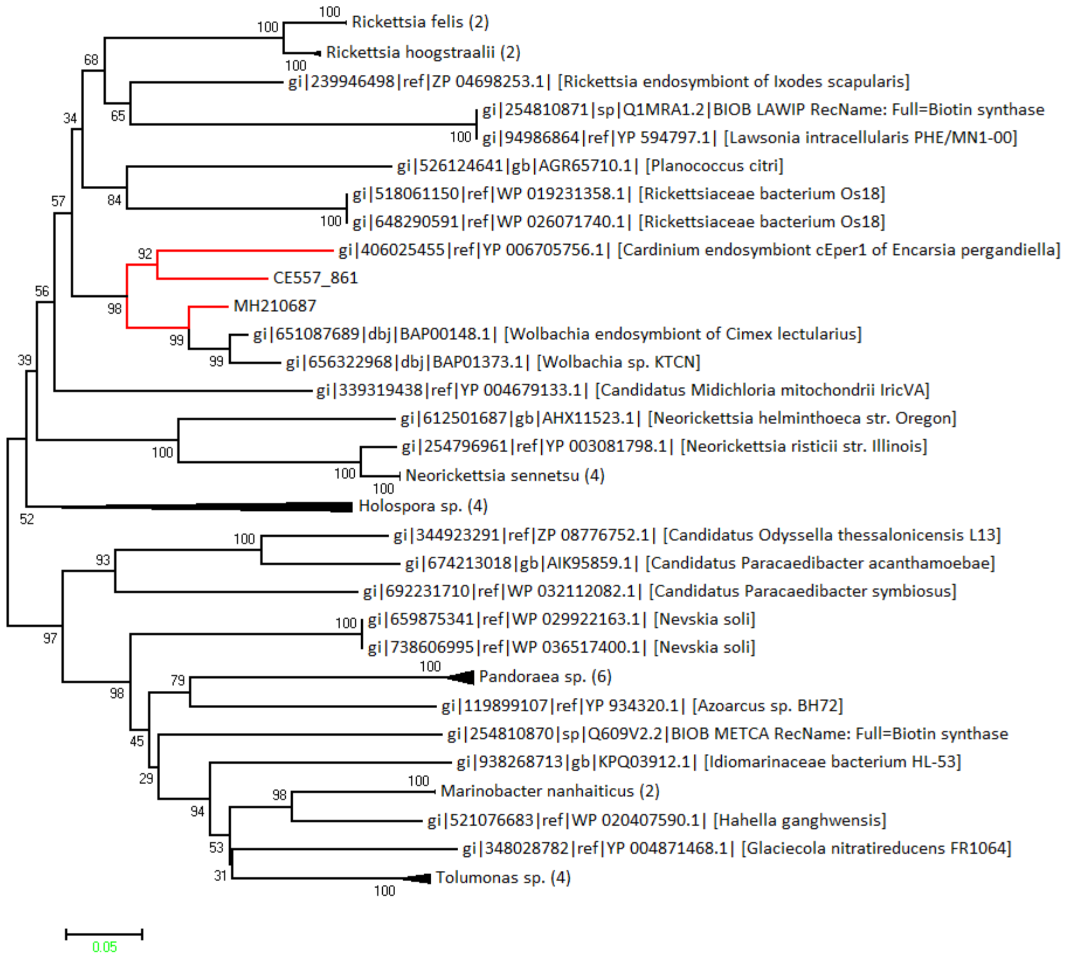

G: CE557_218 (ppdK, pyruvate phosphate dikinase)

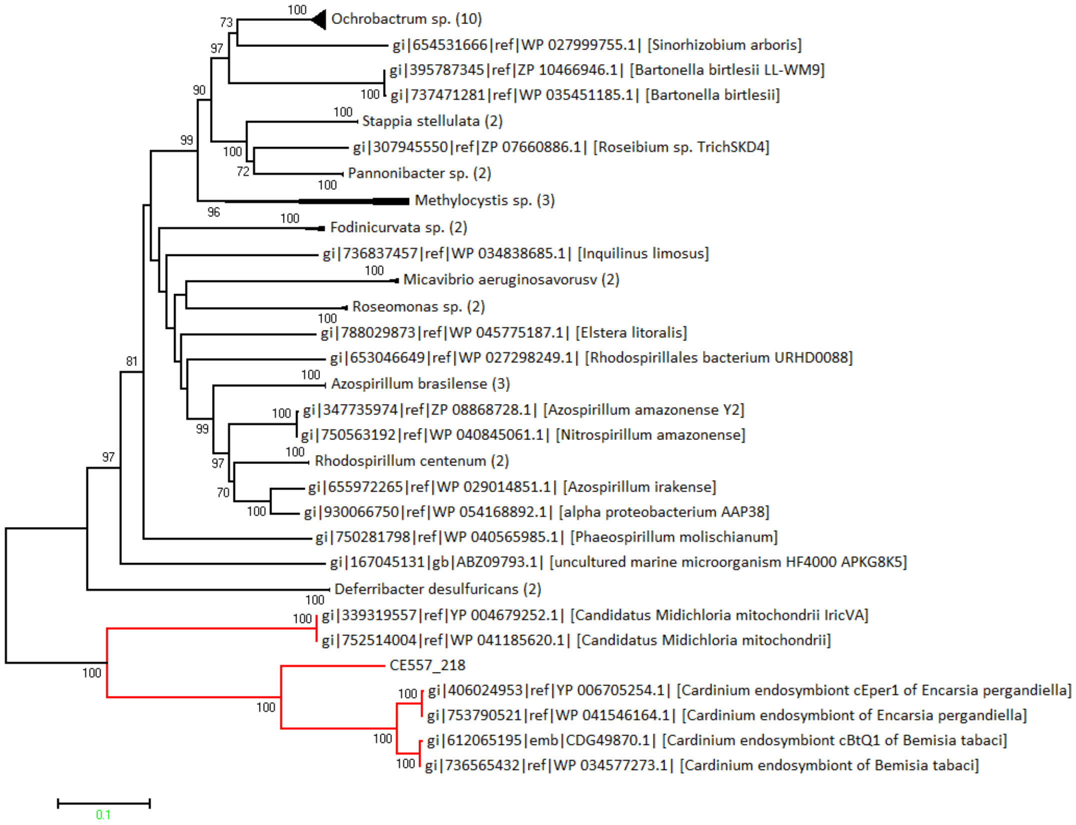

H: CE557_596 (gpml, phosphoglyceromutase)

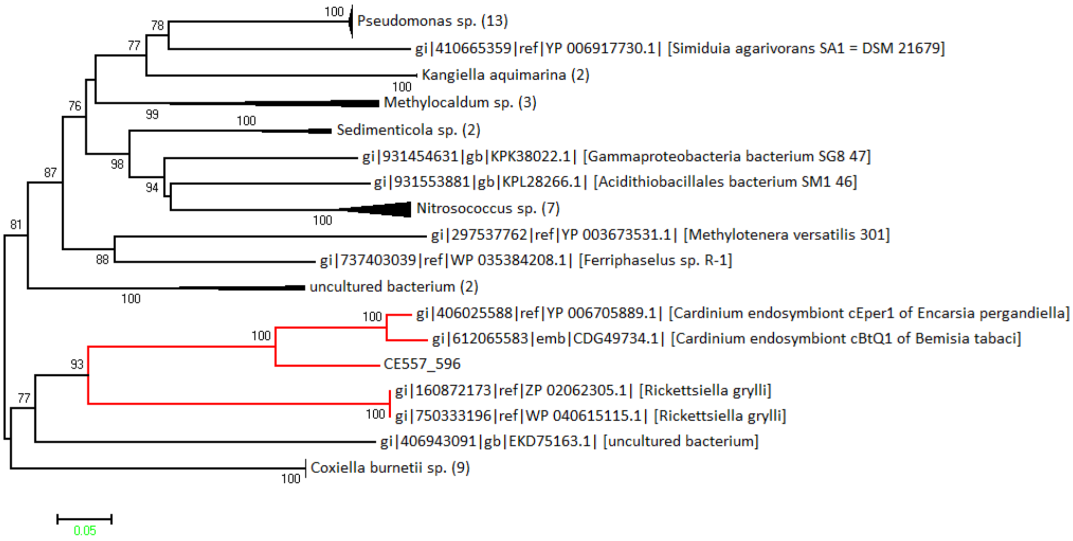

I: CE557_597 (enolase, phosphopyruvate hydratase)

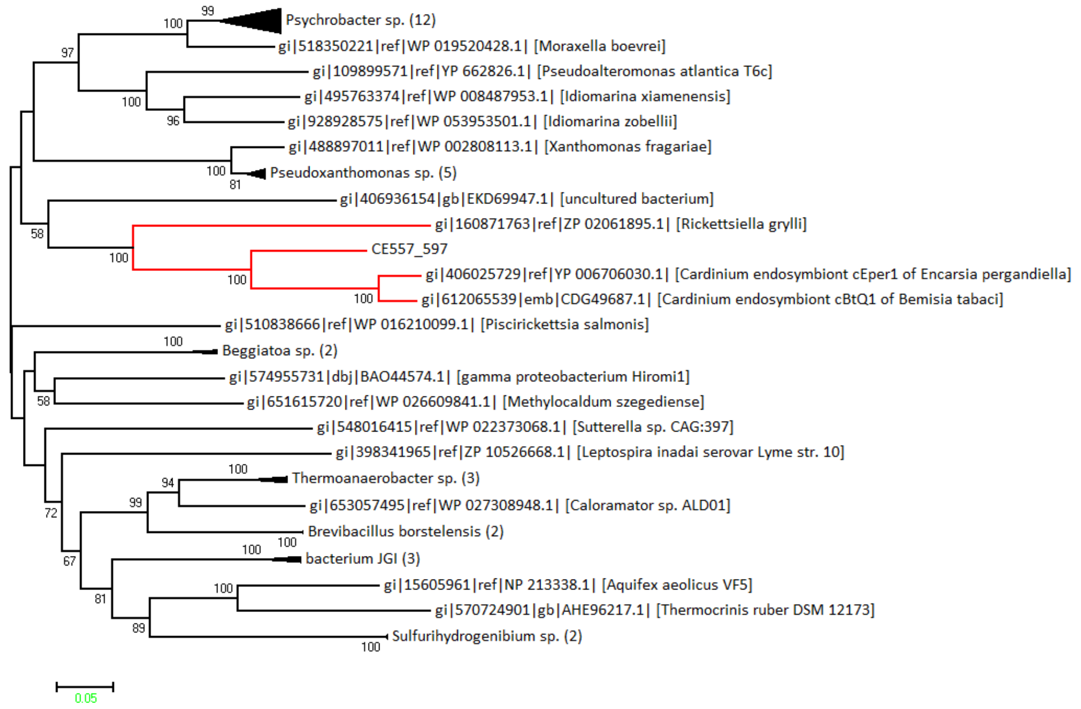

J: CE557_084 (hypothetical protein)

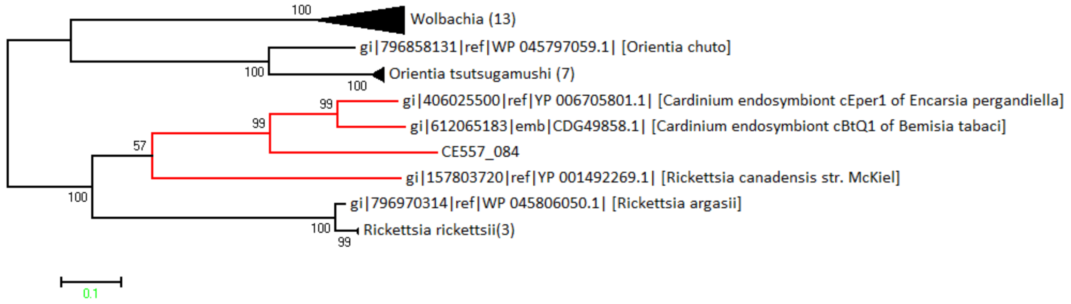

K: CE557_160 (ankyrin-1-like protein)

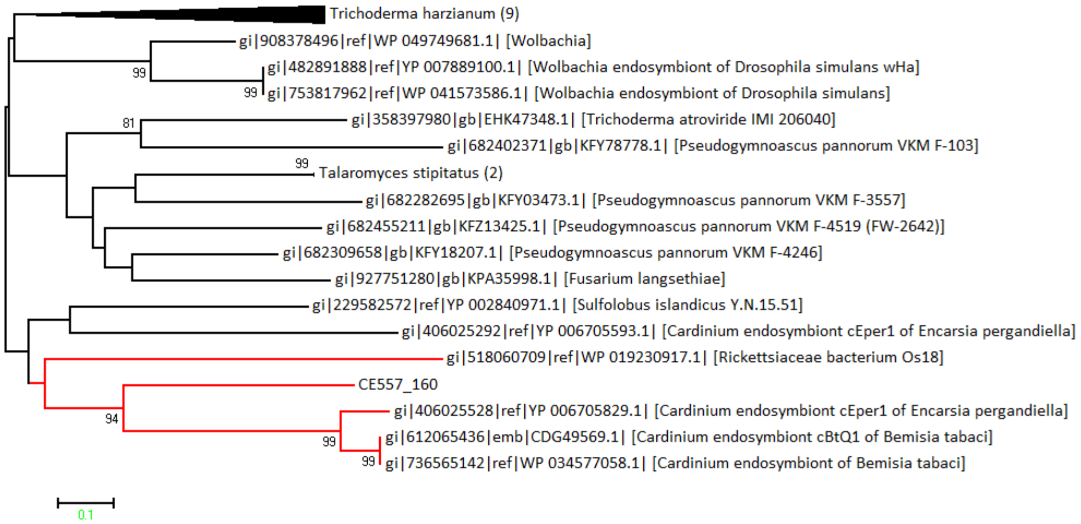

L: CE557_083 (3-methyladenine DNA glycosylase)

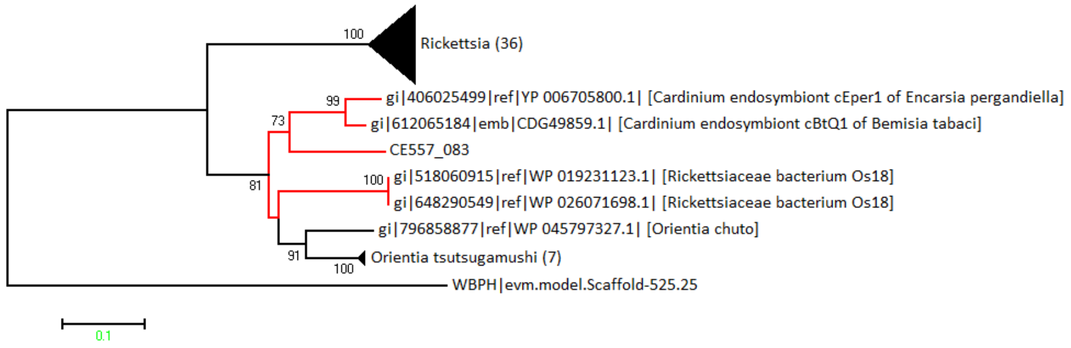

M: CE557_724 (Ankyrin repeat-containing protein)

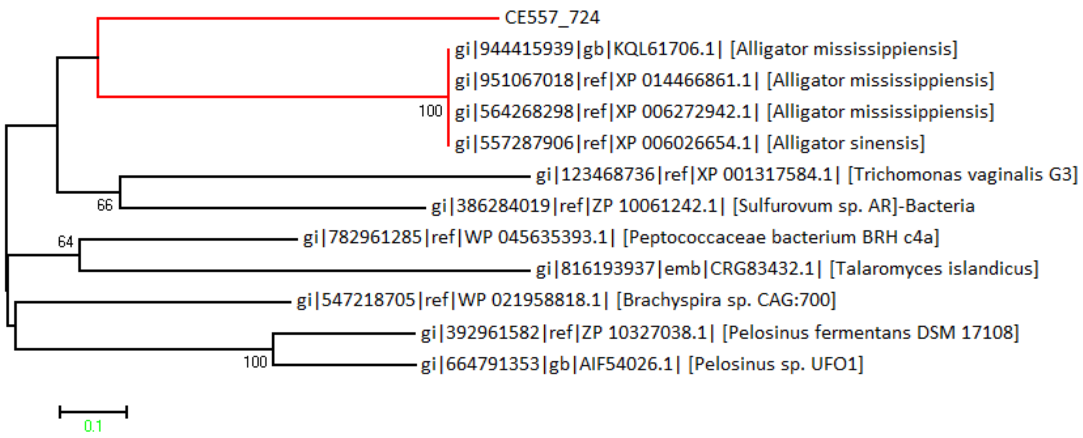

N: CE557_432 (hypothetical protein)

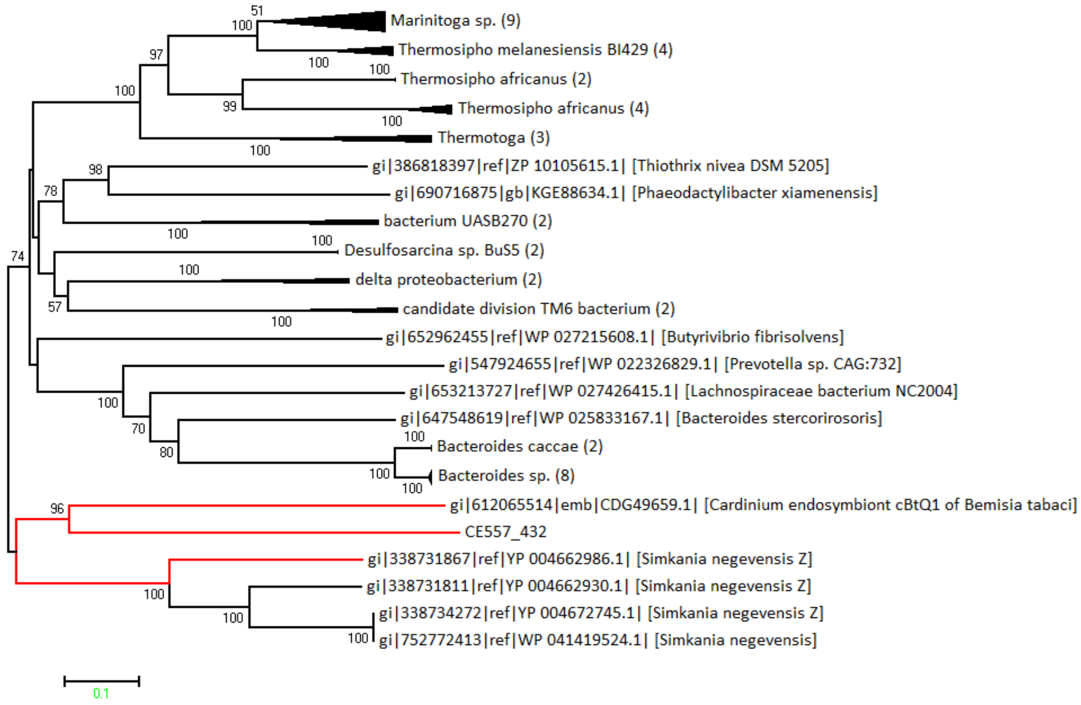

O: CE557_495 (hypothetical protein)

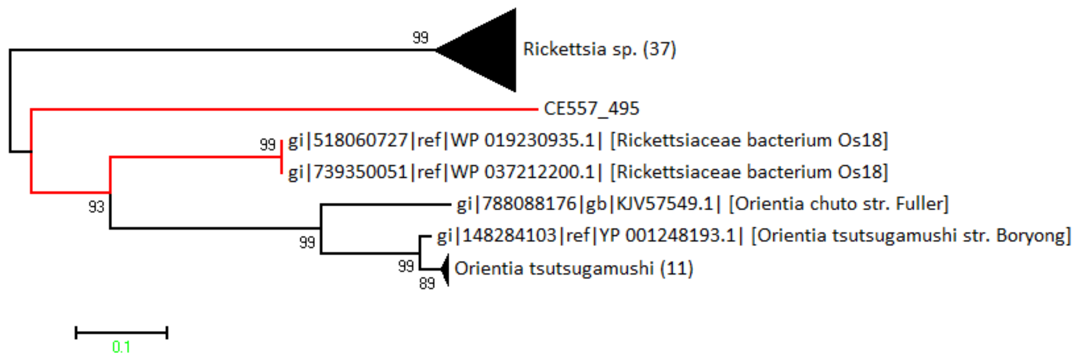

P: CE557_125 (hypothetical protein)

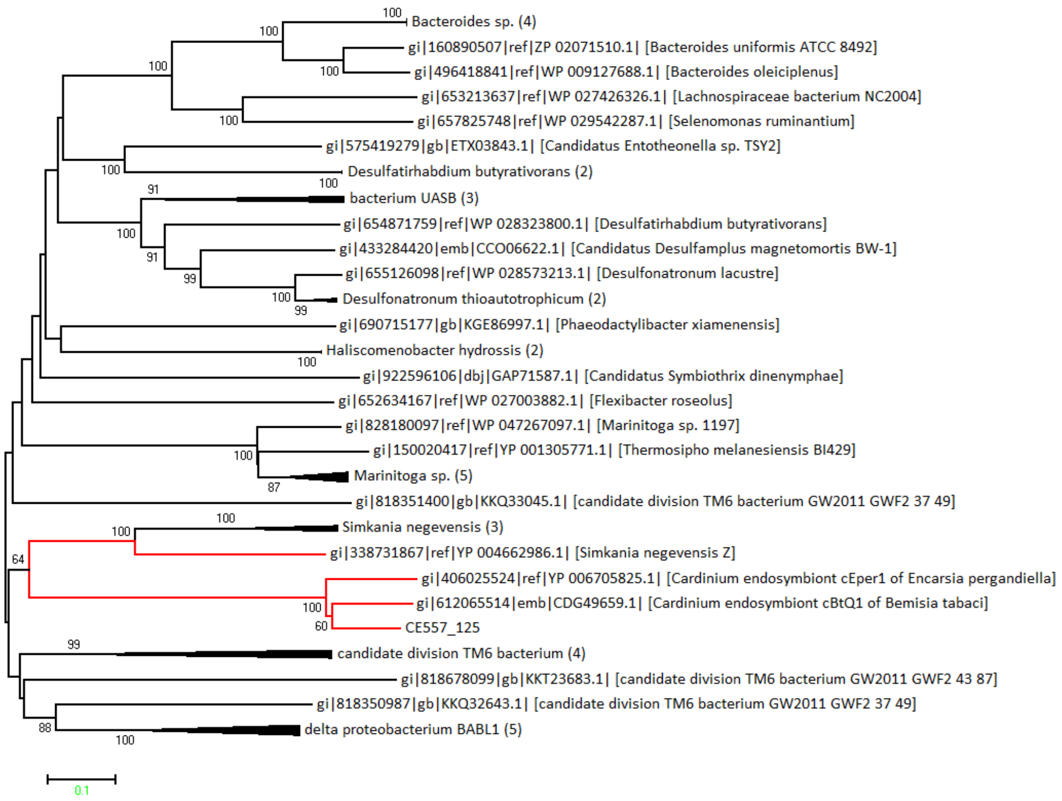

Q: CE557_056 (putative nacht and ankyrin domain protein)

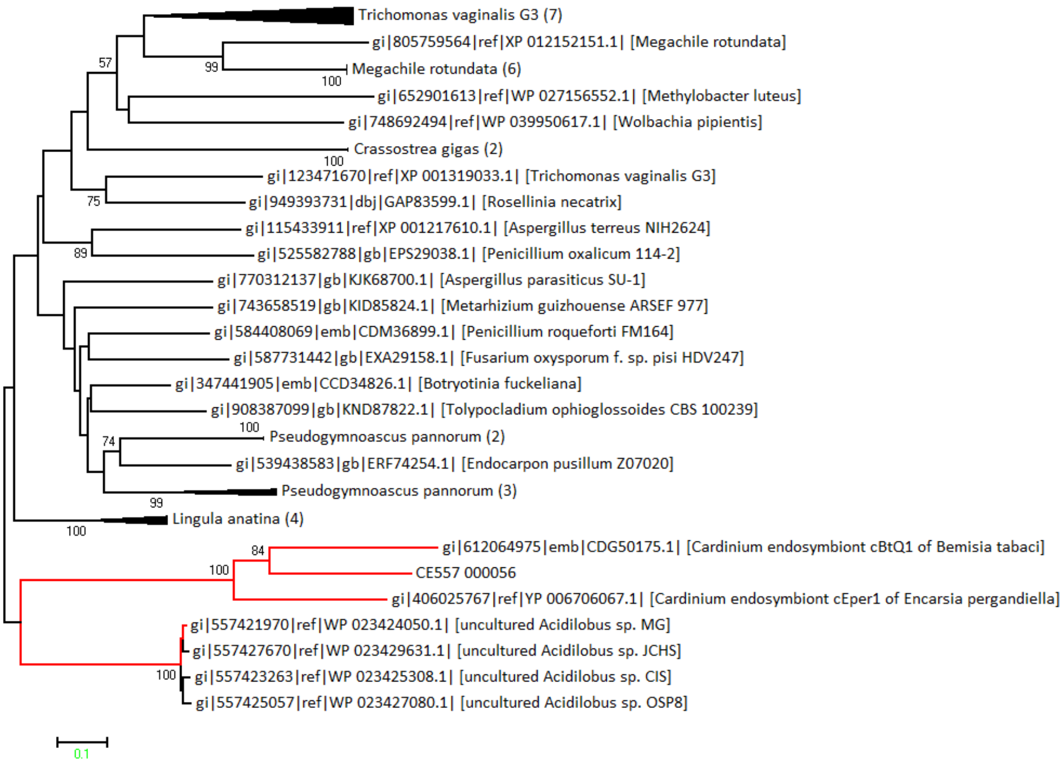

R: CE557_853 (3-demethylubiquinone-9 3-methyltransferase)

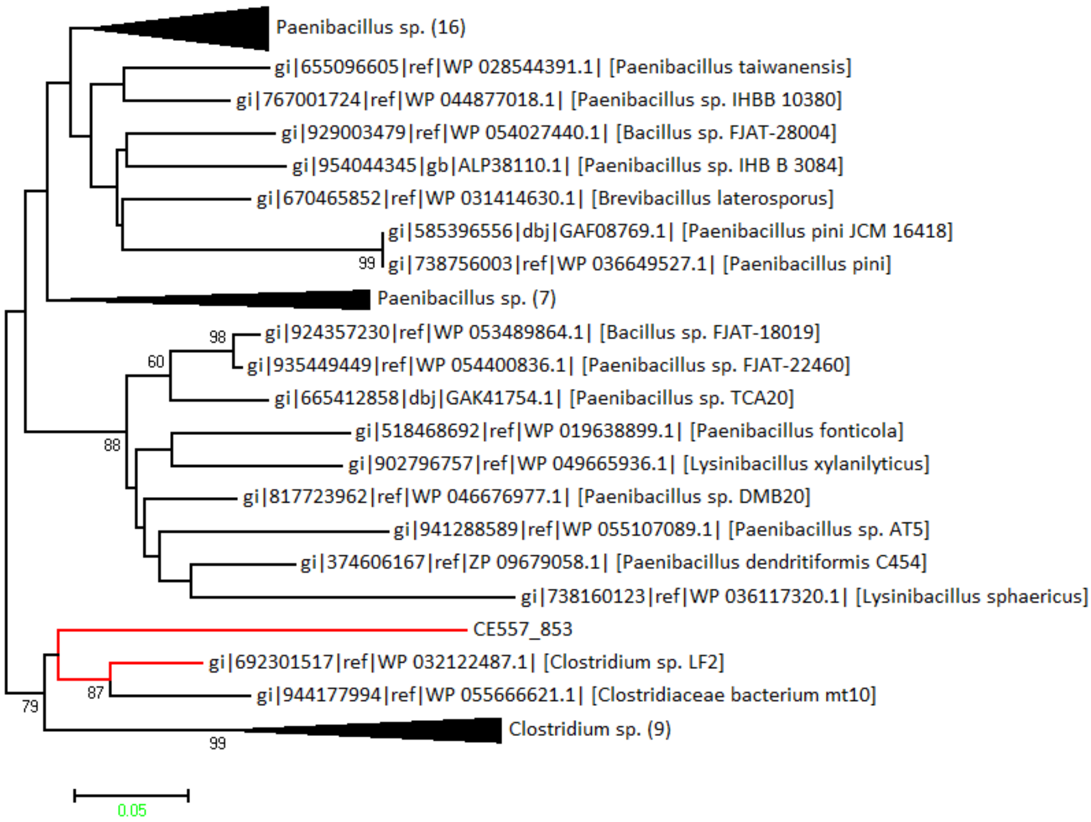

S: CE557_493 (hypothetical protein)

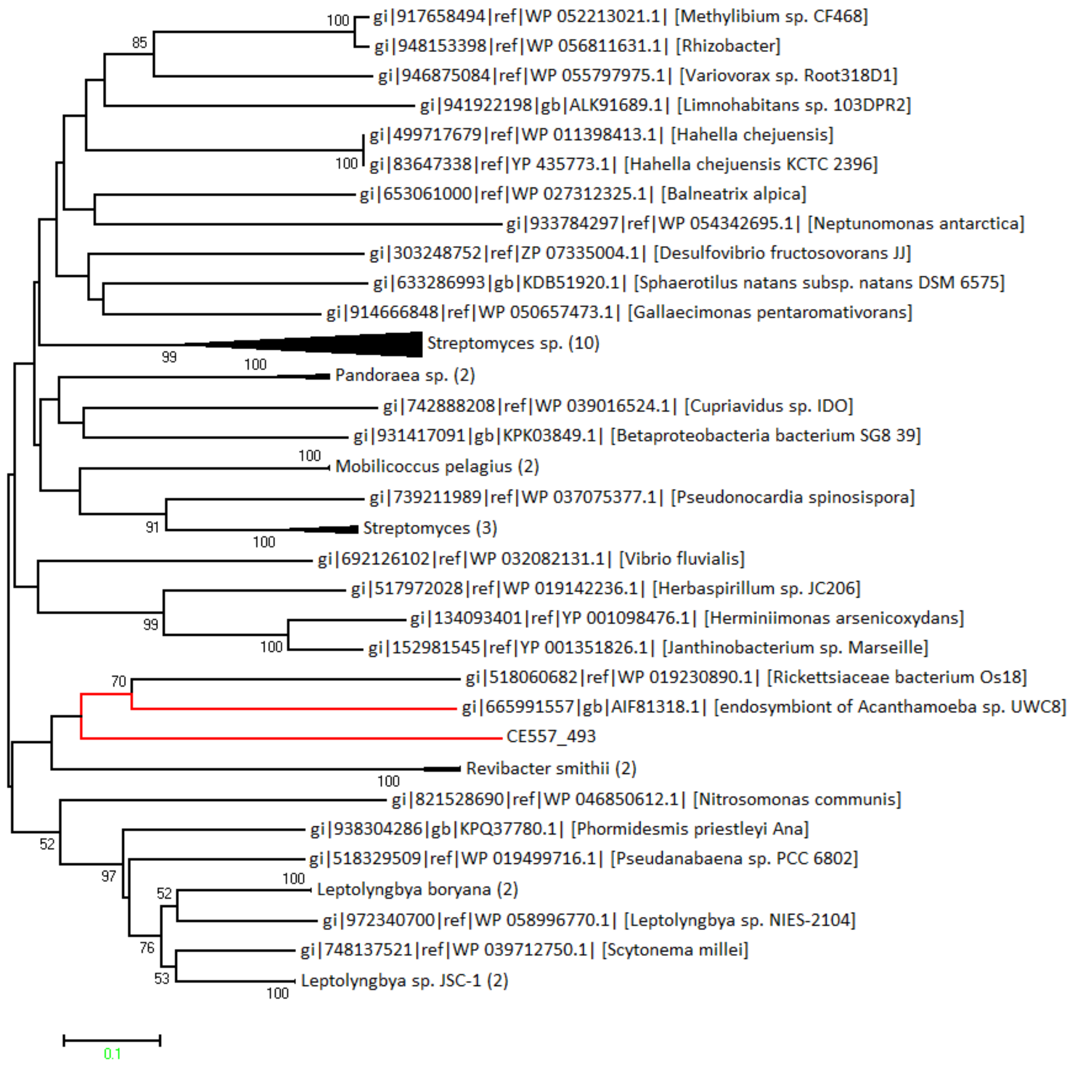

T: CE557_852 (ABC transporter transmembrane region, putative)

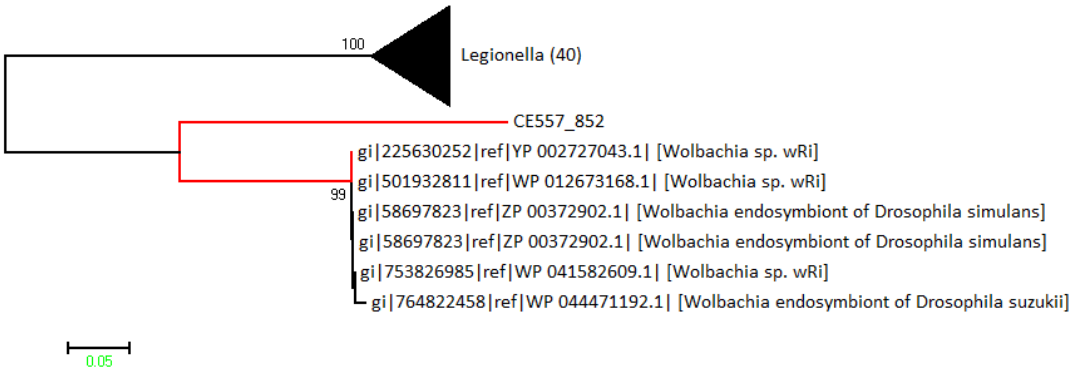

U: CE557_164 (hypothetical protein)

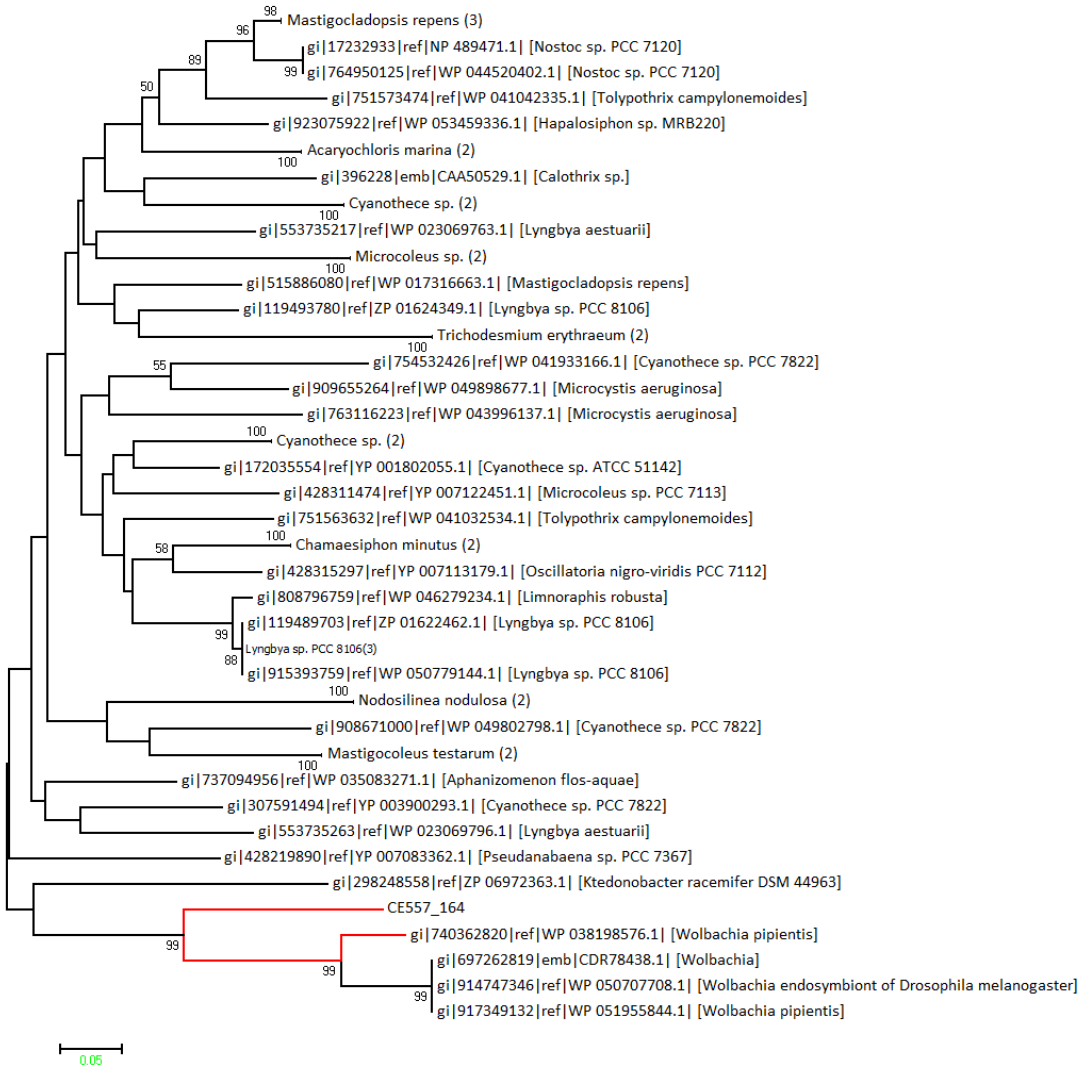

V: CE557_156 (PIF1 helicase)

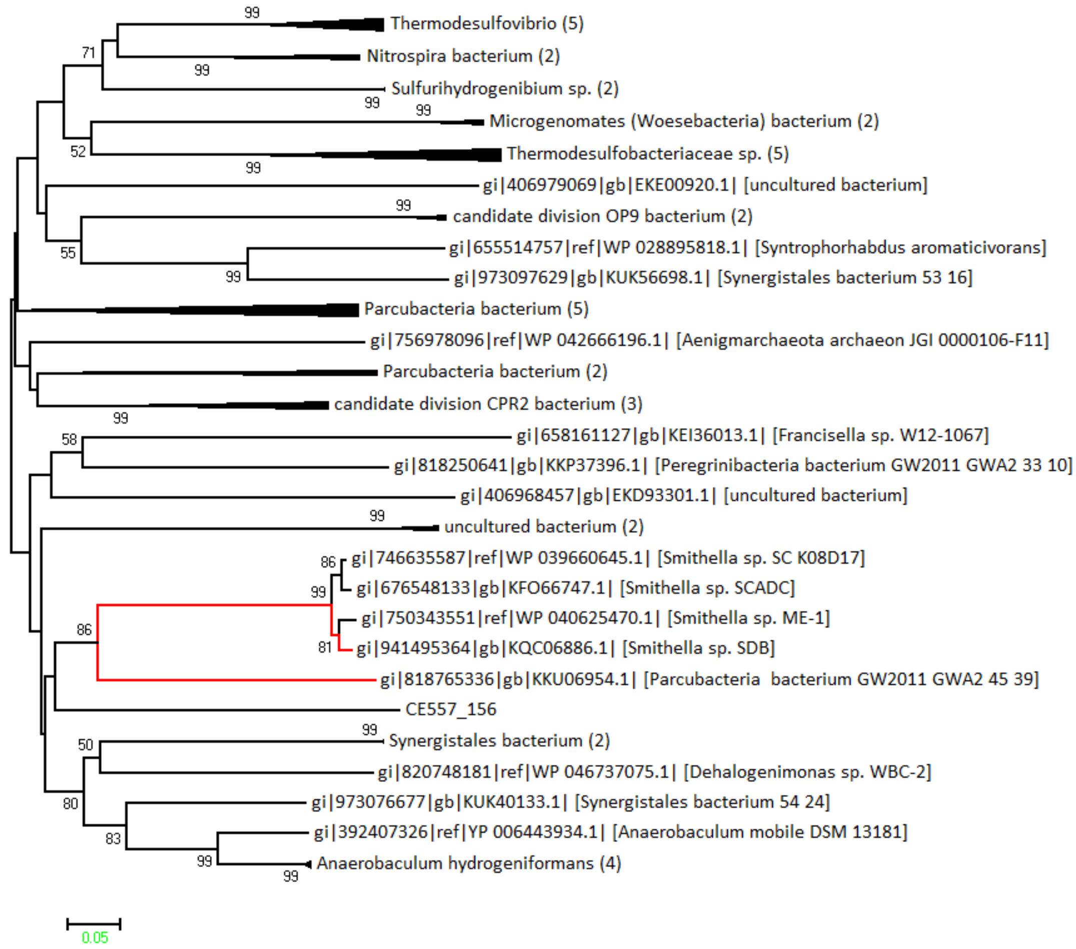

W: CE557_526 (hypothetical protein)

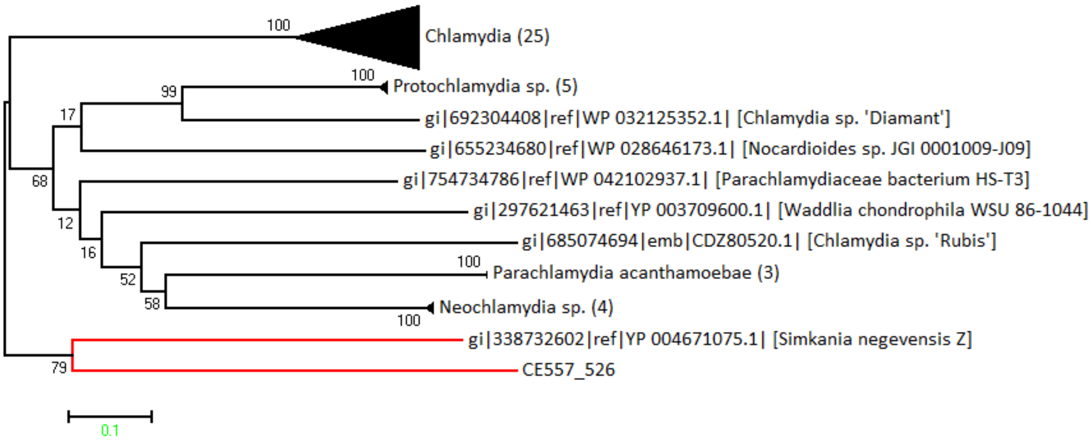

X: CE557_614 (zinc finger protein OZF-like)

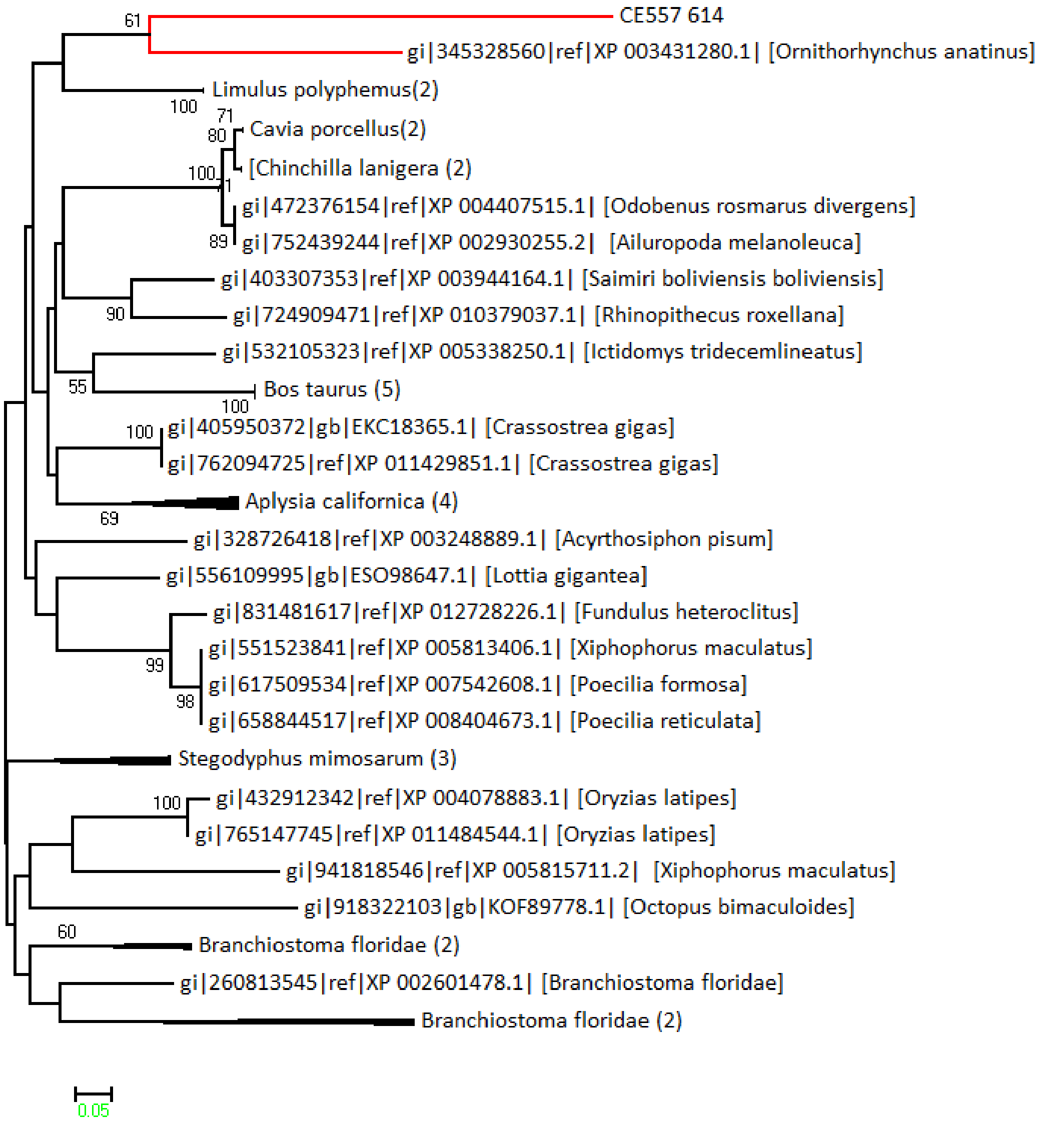

Y: CE557_122 (zinc finger protein 419-like)

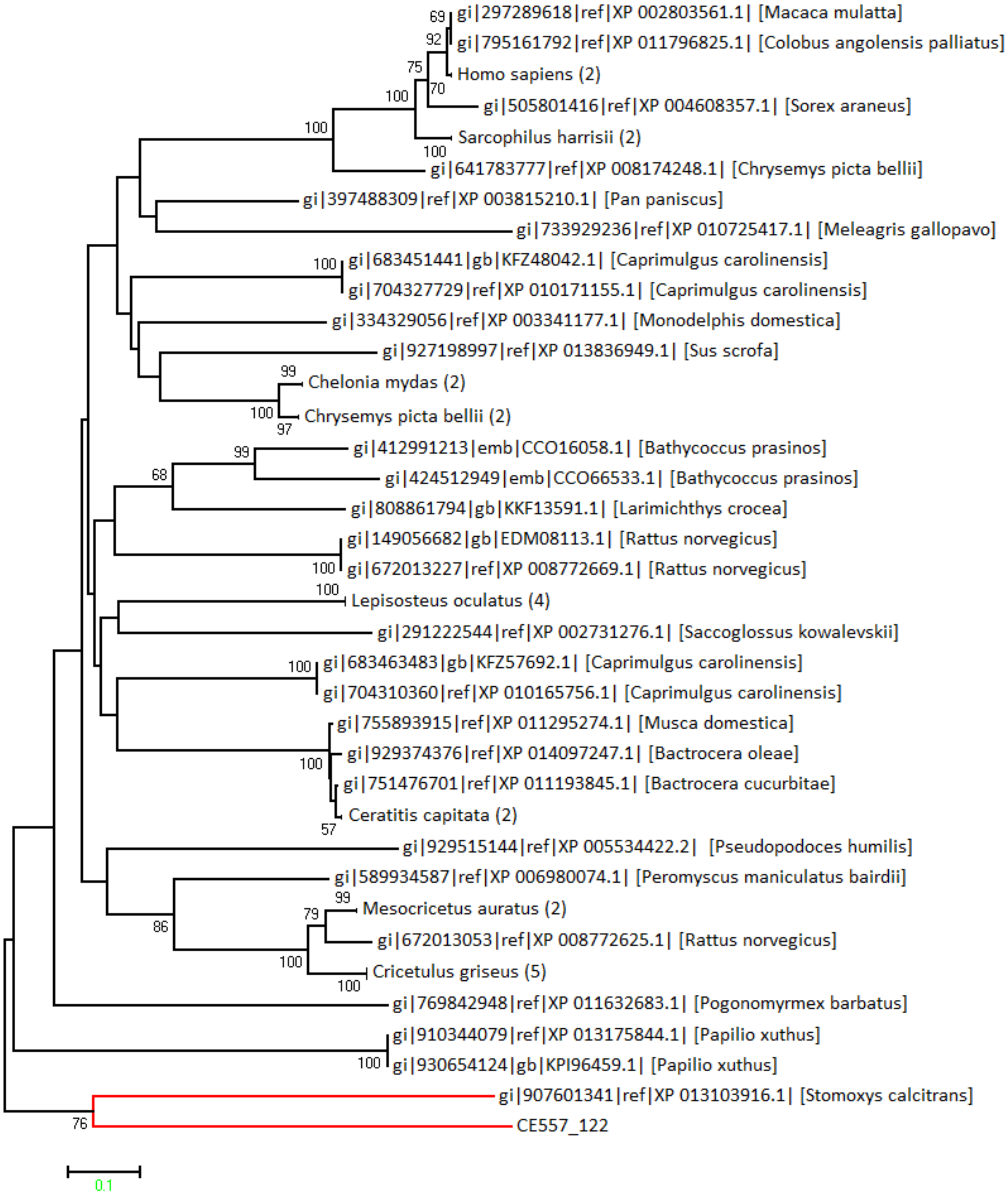

AA: CE557_044 (hypothetical protein)

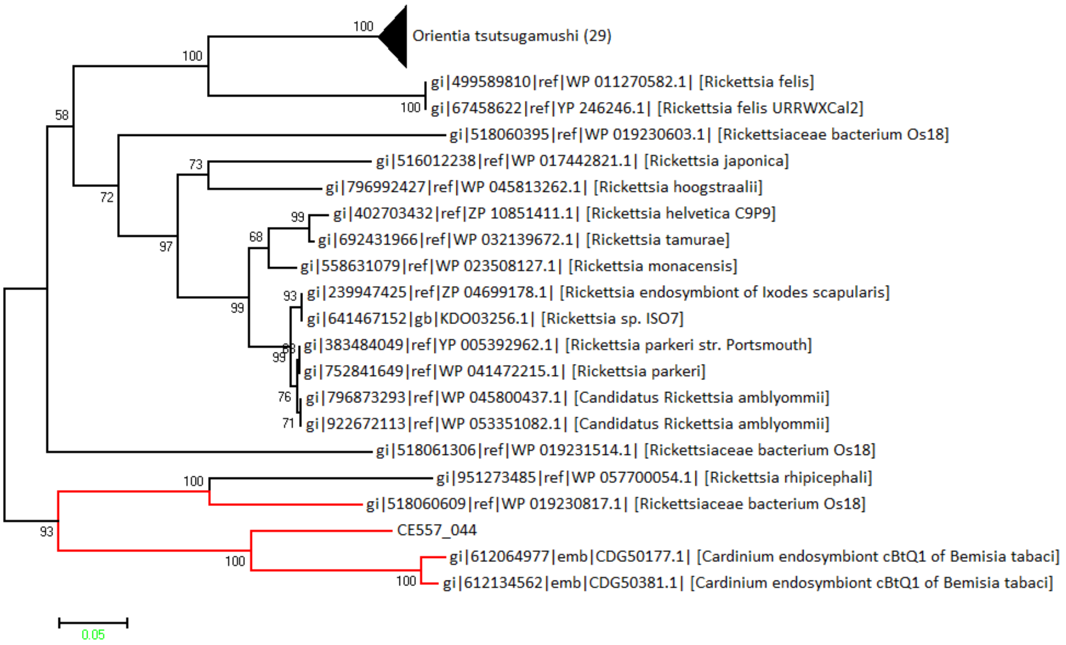

AB: CE557_153 (integrase core domain protein)

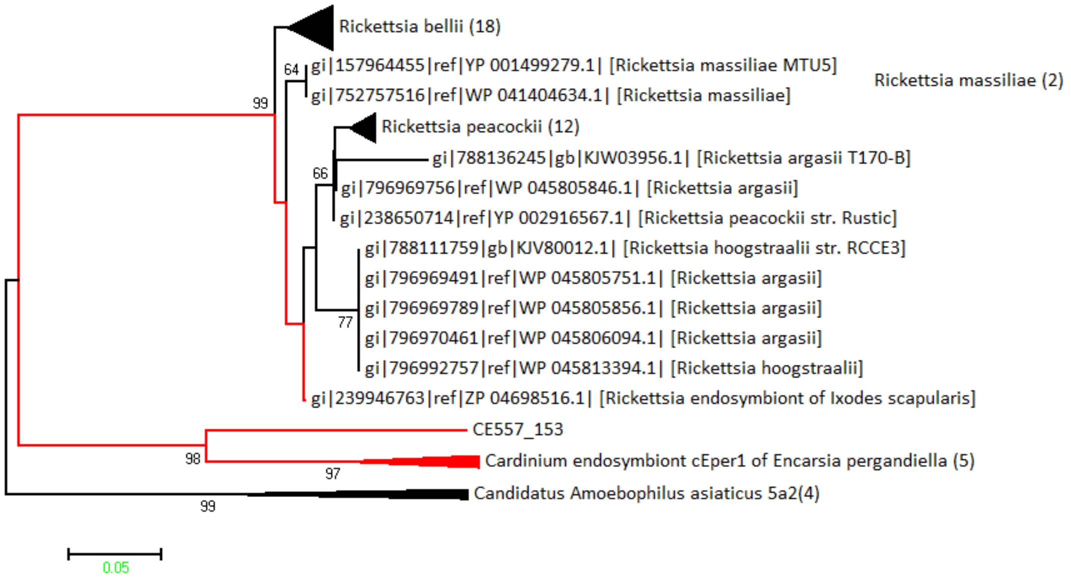

AC: CE557_354 (hypothetical protein)

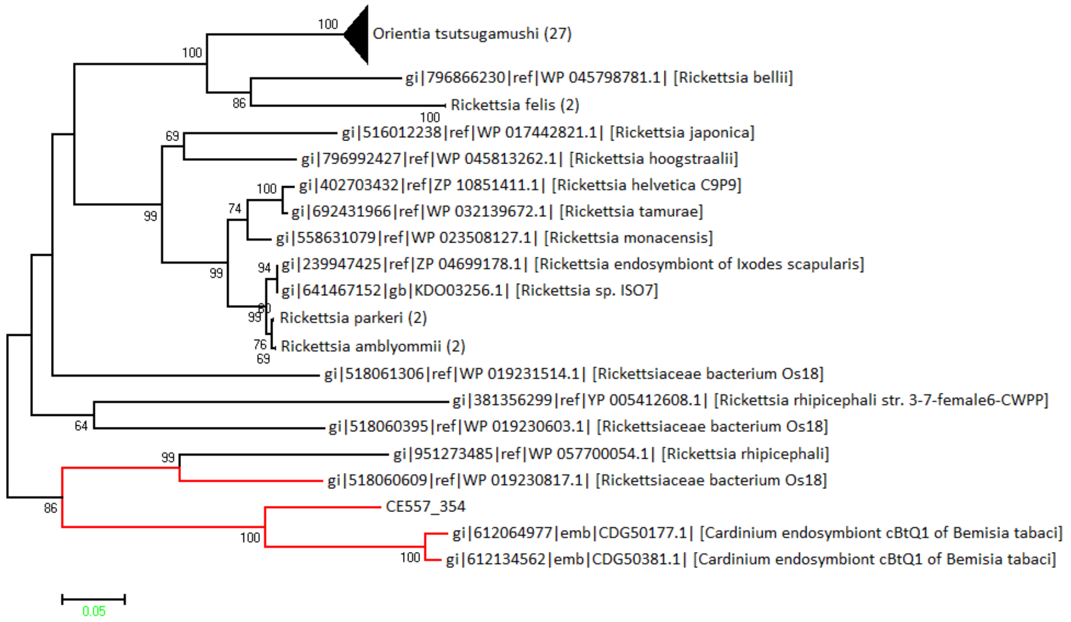

AD: CE557_043 (hypothetical protein)

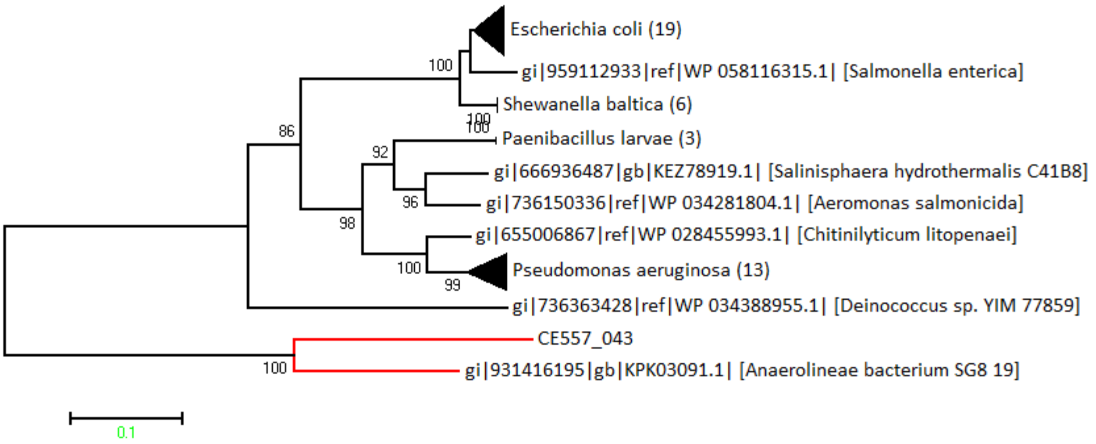

AE: CE557_635 (hypothetical protein)

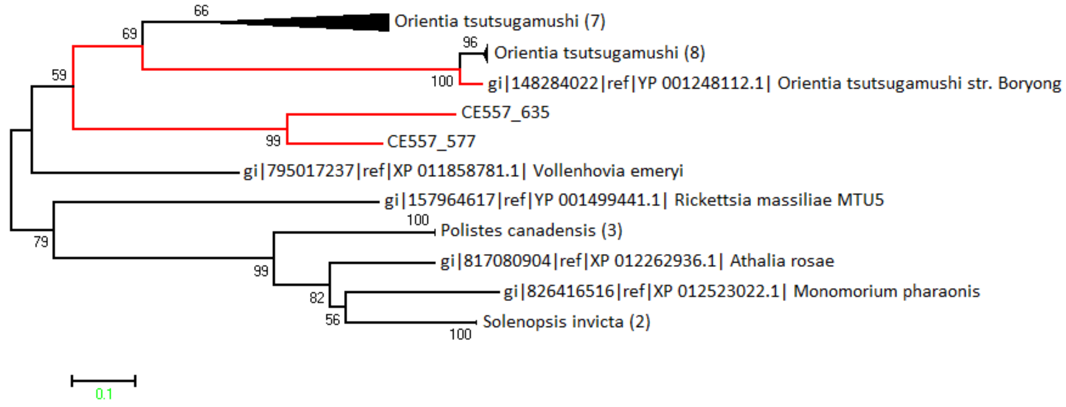

AF: CE557_773 (hypothetical protein)

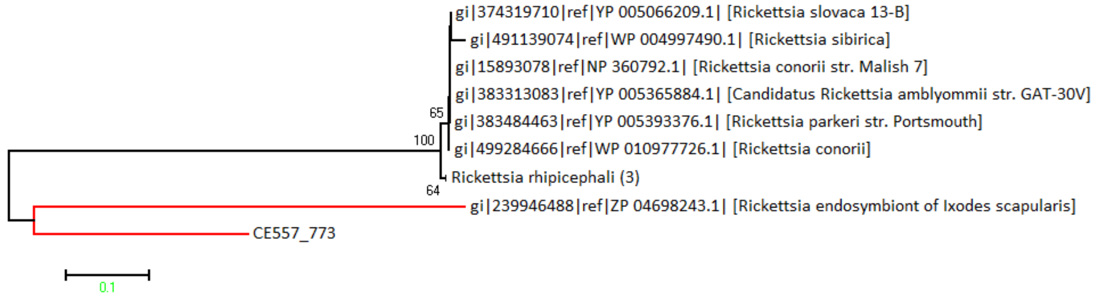

AG: CE557_251/ CE557_448/ CE557_652 (transposase)

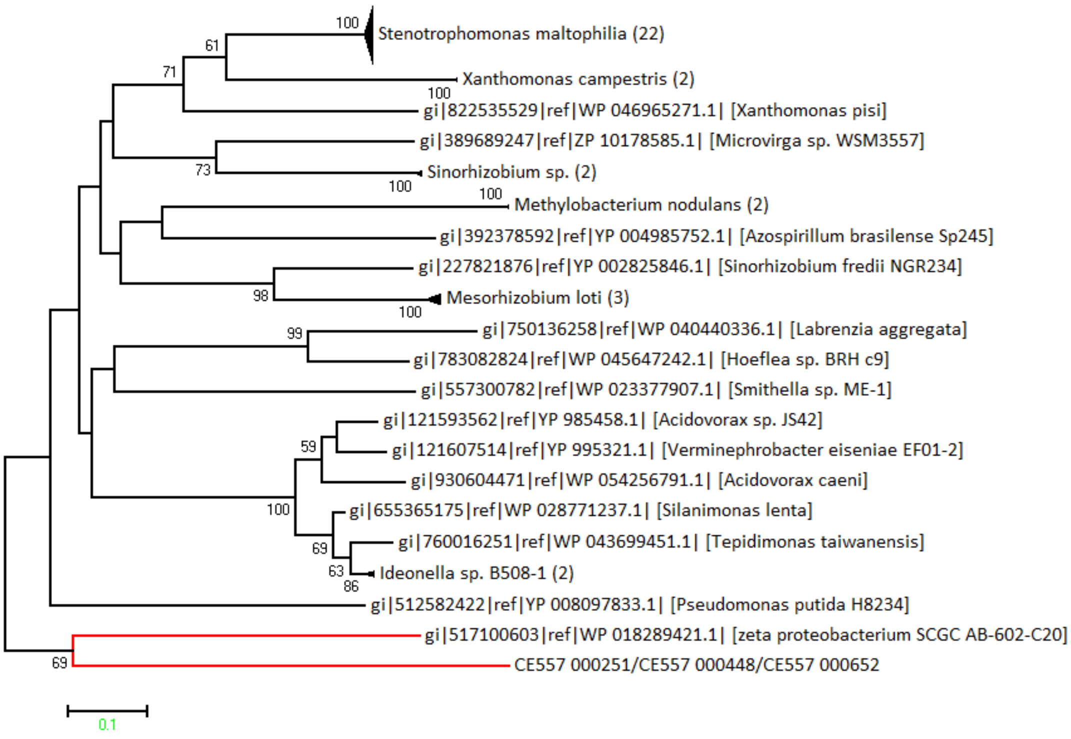

AH: CE557_175/ CE557_234/ CE557_249/ CE557_446/ CE557_650/ CE557_666 (hypothetical protein)

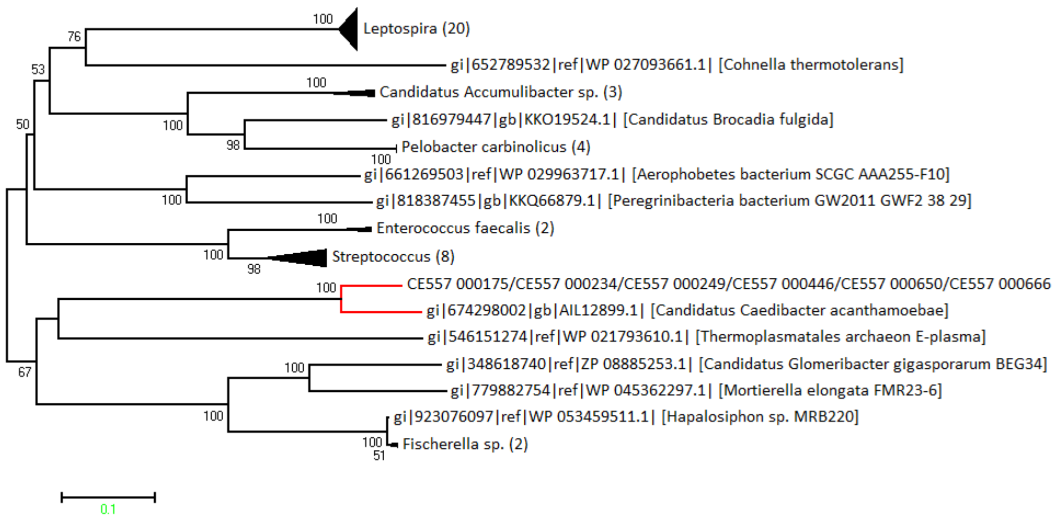

Supplement: Supplementary file 2 — Phylogenetic trees of the genes acquired by the event of HGT in the Cardinium cSfur genome. (PDF 970 kb) [file 12864_2018_5078_MOESM2_ESM.pdf]
