# Supplementary material for: Bacterial endosymbiont Cardinium cSfur genome sequence provides insights for understanding the symbiotic relationship in Sogatella furcifera host
Source: BMC Genomics. 2018 Sep 19;19:688. doi: 10.1186/s12864-018-5078-y (PMC6147030; doi:10.1186/s12864-018-5078-y)
Supplement: Supplementary file 3 — Clusters of orthologous genes (COG) functional classification of the Cardinium cSfur genome (gene homologous cluster number). (PDF 276 kb) [file 12864_2018_5078_MOESM3_ESM.pdf]

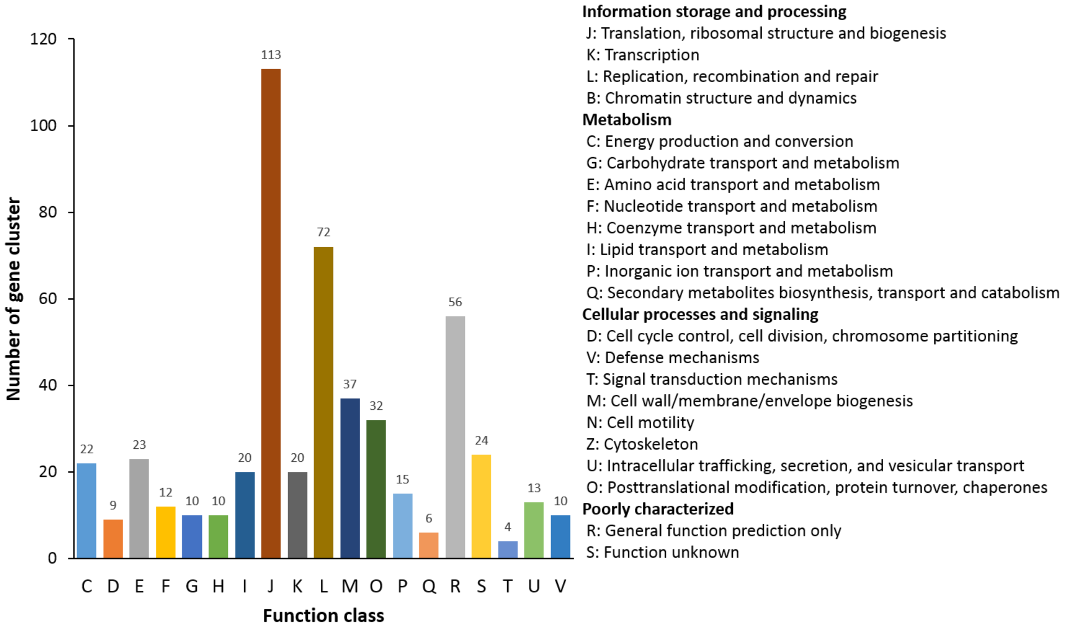

Figure S2: Clusters of orthologous genes (COG) functional classification of the Cardinium cSfur genome (gene homologous clusters number).

795 protein coding genes of the Cardinium cSfur genome classified into 726 homologous gene cluster. Of the 726 gene clusters, 508 were assigned to the COG functional category. The 508 gene clusters classified into the 19 COG functional class.
