# Supplementary material for: Bacterial endosymbiont Cardinium cSfur genome sequence provides insights for understanding the symbiotic relationship in Sogatella furcifera host
Source: BMC Genomics. 2018 Sep 19;19:688. doi: 10.1186/s12864-018-5078-y (PMC6147030; doi:10.1186/s12864-018-5078-y)
Supplement: Supplementary file 8 — Pathway statistics of Cardinium cSf. (PDF 85 kb) [file 12864_2018_5078_MOESM8_ESM.pdf]

Additional file 3:

1. Pathway statistics of *Cardinium* cSfur

Complete pathways:

C 00010 Glycolysis [PATH:ko00010]

C 00550 Peptidoglycan biosynthesis [PATH:ko00550]

C 00780 Biotin metabolism [PATH:ko00780]

C 00785 Lipoic acid metabolism [PATH:ko00785]

Incomplete pathways:

C 00061 Fatty acid biosynthesis [PATH:ko00061]

C 00230 Purine metabolism [PATH:ko00230]

C 00240 Pyrimidine metabolism [PATH:ko00240]

C 00010 Gluconeogenesis [PATH:ko00010]

C 00051 Fructose and mannose metabolism [PATH:ko00051]

C 00500 Starch and sucrose metabolism [PATH:ko00500]

C 00520 Amino sugar and nucleotide sugar metabolism [PATH:ko00520]

C 00620 Pyruvate metabolism [PATH:ko00620]

C 00630 Glyoxylate and dicarboxylate metabolism [PATH:ko00630]

C 00640 Propanoate metabolism [PATH:ko00640]

C 00650 Butanoate metabolism [PATH:ko00650]

C 00562 Inositol phosphate metabolism [PATH:ko00562]

C 00190 Oxidative phosphorylation [PATH:ko00190]

C 00195 Photosynthesis [PATH:ko00195]

C 00710 Carbon fixation in photosynthetic organisms [PATH:ko00710]

C 00720 Carbon fixation pathways in prokaryotes [PATH:ko00720]

C 00680 Methane metabolism [PATH:ko00680]

C 00910 Nitrogen metabolism [PATH:ko00910]

C 00920 Sulfur metabolism [PATH:ko00920]

C 00071 Fatty acid degradation [PATH:ko00071]

C 00561 Glycerolipid metabolism [PATH:ko00561]

C 00564 Glycerophospholipid metabolism [PATH:ko00564]

C 00565 Ether lipid metabolism [PATH:ko00565]

C 01040 Biosynthesis of unsaturated fatty acids [PATH:ko01040]

C 00590 Arachidonic acid metabolism [PATH:ko00590]

C 00260 Glycine, serine and threonine metabolism [PATH:ko00260]

C 00300 Lysine biosynthesis [PATH:ko00300]

C 00450 Selenocompound metabolism [PATH:ko00450]

C 00460 Cyanoamino acid metabolism [PATH:ko00460]

C 00471 D-Glutamine and D-glutamate metabolism [PATH:ko00471]

C 00473 D-Alanine metabolism [PATH:ko00473]

C 00480 Glutathione metabolism [PATH:ko00480]

C 00540 Lipopolysaccharide biosynthesis [PATH:ko00540]

C 00790 Folate biosynthesis [PATH:ko00790]

C 00670 One carbon pool by folate [PATH:ko00670]
C 00860 Porphyrin and chlorophyll metabolism [PATH:ko00860]
C 00253 Tetracycline biosynthesis [PATH:ko00253]
C 00523 Polyketide sugar unit biosynthesis [PATH:ko00523]
C 00521 Streptomycin biosynthesis [PATH:ko00521]

Nonexistent pathways:
C 00020 Citrate cycle (TCA cycle) [PATH:ko00020]
C 00030 Pentose phosphate pathway [PATH:ko00030]
C 00040 Pentose and glucuronate interconversions [PATH:ko00040]
C 00052 Galactose metabolism [PATH:ko00052]
C 00053 Ascorbate and aldarate metabolism [PATH:ko00053]
C 00660 C5-Branched dibasic acid metabolism [PATH:ko00660]
C 00196 Photosynthesis - antenna proteins [PATH:ko00196]
C 00194 Photosynthesis proteins [BR:ko00194]
C 00062 Fatty acid elongation [PATH:ko00062]
C 00072 Synthesis and degradation of ketone bodies [PATH:ko00072]
C 00073 Cutin, suberine and wax biosynthesis [PATH:ko00073]
C 00100 Steroid biosynthesis [PATH:ko00100]
C 00120 Primary bile acid biosynthesis [PATH:ko00120]
C 00121 Secondary bile acid biosynthesis [PATH:ko00121]
C 00140 Steroid hormone biosynthesis [PATH:ko00140]
C 00600 Sphingolipid metabolism [PATH:ko00600]
C 00591 Linoleic acid metabolism [PATH:ko00591]
C 00592 alpha-Linolenic acid metabolism [PATH:ko00592]
C 01004 Lipid biosynthesis proteins [BR:ko01004]
C 00250 Alanine, aspartate and glutamate metabolism [PATH:ko00250]
C 00270 Cysteine and methionine metabolism [PATH:ko00270]
C 00280 Valine, leucine and isoleucine degradation [PATH:ko00280]
C 00290 Valine, leucine and isoleucine biosynthesis [PATH:ko00290]
C 00310 Lysine degradation [PATH:ko00310]
C 00220 Arginine biosynthesis [PATH:ko00220]
C 00330 Arginine and proline metabolism [PATH:ko00330]
C 00340 Histidine metabolism [PATH:ko00340]
C 00350 Tyrosine metabolism [PATH:ko00350]
C 00360 Phenylalanine metabolism [PATH:ko00360]
C 00380 Tryptophan metabolism [PATH:ko00380]
C 00400 Phenylalanine, tyrosine and tryptophan biosynthesis [PATH:ko00400]
C 01007 Amino acid related enzymes [BR:ko01007]
C 00410 beta-Alanine metabolism [PATH:ko00410]
C 00430 Taurine and hypotaurine metabolism [PATH:ko00430]
C 00440 Phosphonate and phosphinate metabolism [PATH:ko00440]
C 00472 D-Arginine and D-ornithine metabolism [PATH:ko00472]
C 01003 Glycosyltransferases [BR:ko01003]

C 00510 N-Glycan biosynthesis [PATH:ko00510]
C 00513 Various types of N-glycan biosynthesis [PATH:ko00513]
C 00512 Mucin type O-glycan biosynthesis [PATH:ko00512]
C 00514 Other types of O-glycan biosynthesis [PATH:ko00514]
C 00532 Glycosaminoglycan biosynthesis-chondroitin sulfate [PATH:ko00532]
C 00534 Glycosaminoglycan biosynthesis - heparan sulfate / heparin [PATH:ko00534]
C 00533 Glycosaminoglycan biosynthesis - keratan sulfate [PATH:ko00533]
C 00535 Proteoglycans [BR:ko00535]
C 00536 Glycosaminoglycan binding proteins [BR:ko00536]
C 00537 Glycosylphosphatidylinositol (GPI)-anchored proteins [BR:ko00537]
C 00531 Glycosaminoglycan degradation [PATH:ko00531]
C 00563 Glycosylphosphatidylinositol(GPI)-anchor biosynthesis [PATH:ko00563]
C 00601 Glycosphingolipid biosynthesis - lacto and neolacto series [PATH:ko00601]
C 00603 Glycosphingolipid biosynthesis - globo series [PATH:ko00603]
C 00604 Glycosphingolipid biosynthesis - ganglio series [PATH:ko00604]
C 01005 Lipopolysaccharide biosynthesis proteins [BR:ko01005]
C 00730 Thiamine metabolism [PATH:ko00730]
C 00740 Riboflavin metabolism [PATH:ko00740]
C 00750 Vitamin B6 metabolism [PATH:ko00750]
C 00760 Nicotinate and nicotinamide metabolism [PATH:ko00760]
C 00770 Pantothenate and CoA biosynthesis [PATH:ko00770]
C 00830 Retinol metabolism [PATH:ko00830]
C 00130 Ubiquinone and other terpenoid-quinone biosynthesis [PATH:ko00130]
C 01006 Prenyltransferases [BR:ko01006]
C 00900 Terpenoid backbone biosynthesis [PATH:ko00900]
C 00902 Monoterpenoid biosynthesis [PATH:ko00902]
C 00909 Sesquiterpenoid and triterpenoid biosynthesis [PATH:ko00909]
C 00904 Diterpenoid biosynthesis [PATH:ko00904]
C 00906 Carotenoid biosynthesis [PATH:ko00906]
C 00905 Brassinosteroid biosynthesis [PATH:ko00905]
C 00981 Insect hormone biosynthesis [PATH:ko00981]
C 00908 Zeatin biosynthesis [PATH:ko00908]
C 00903 Limonene and pinene degradation [PATH:ko00903]
C 00281 Geraniol degradation [PATH:ko00281]
C 01008 Polyketide biosynthesis proteins [BR:ko01008]
C 01052 Type I polyketide structures [PATH:ko01052]
C 00522 Biosynthesis of 12-, 14- and 16-membered macrolides [PATH:ko00522]
C 01051 Biosynthesis of ansamycins [PATH:ko01051]
C 01056 Biosynthesis of type II polyketide backbone [PATH:ko01056]
C 01057 Biosynthesis of type II polyketide products [PATH:ko01057]
C 01054 Nonribosomal peptide structures [PATH:ko01054]
C 01053 Biosynthesis of siderophore group nonribosomal peptides [PATH:ko01053]
C 01055 Biosynthesis of vancomycin group antibiotics [PATH:ko01055]
C 00940 Phenylpropanoid biosynthesis [PATH:ko00940]

C 00945 Stilbenoid, diarylheptanoid and gingerol biosynthesis [PATH:ko00945]
C 00941 Flavonoid biosynthesis [PATH:ko00941]
C 00944 Flavone and flavonol biosynthesis [PATH:ko00944]
C 00942 Anthocyanin biosynthesis [PATH:ko00942]
C 00943 Isoflavonoid biosynthesis [PATH:ko00943]
C 00901 Indole alkaloid biosynthesis [PATH:ko00901]
C 00403 Indole diterpene alkaloid biosynthesis [PATH:ko00403]
C 00950 Isoquinoline alkaloid biosynthesis [PATH:ko00950]
C 00960 Tropane, piperidine and pyridine alkaloid biosynthesis [PATH:ko00960]
C 01058 Acridone alkaloid biosynthesis [PATH:ko01058]
C 00232 Caffeine metabolism [PATH:ko00232]
C 00965 Betalain biosynthesis [PATH:ko00965]
C 00966 Glucosinolate biosynthesis [PATH:ko00966]
C 00402 Benzoxazinoid biosynthesis [PATH:ko00402]
C 00311 Penicillin and cephalosporin biosynthesis [PATH:ko00311]
C 00332 Carbapenem biosynthesis [PATH:ko00332]
C 00261 Monobactam biosynthesis [PATH:ko00261]
C 00331 Clavulanic acid biosynthesis [PATH:ko00331]
C 00524 Butirosin and neomycin biosynthesis [PATH:ko00524]
C 00231 Puromycin biosynthesis [PATH:ko00231]
C 00401 Novobiocin biosynthesis [PATH:ko00401]
C 00404 Staurosporine biosynthesis [PATH:ko00404]
C 00254 Aflatoxin biosynthesis [PATH:ko00254]
C 00362 Benzoate degradation [PATH:ko00362]
C 00627 Aminobenzoate degradation [PATH:ko00627]
C 00364 Fluorobenzoate degradation [PATH:ko00364]
C 00625 Chloroalkane and chloroalkene degradation [PATH:ko00625]
C 00361 Chlorocyclohexane and chlorobenzene degradation [PATH:ko00361]
C 00623 Toluene degradation [PATH:ko00623]
C 00622 Xylene degradation [PATH:ko00622]
C 00633 Nitrotoluene degradation [PATH:ko00633]
C 00642 Ethylbenzene degradation [PATH:ko00642]
C 00643 Styrene degradation [PATH:ko00643]
C 00791 Atrazine degradation [PATH:ko00791]
C 00930 Caprolactam degradation [PATH:ko00930]
C 00351 1,1,1-Trichloro-2,2-bis(4-chlorophenyl)ethane (DDT) degradation [PATH:ko00351]
C 00363 Bisphenol degradation [PATH:ko00363]
C 00621 Dioxin degradation [PATH:ko00621]
C 00626 Naphthalene degradation [PATH:ko00626]
C 00624 Polycyclic aromatic hydrocarbon degradation [PATH:ko00624]
C 00365 Furfural degradation [PATH:ko00365]
C 00984 Steroid degradation [PATH:ko00984]
C 00980 Metabolism of xenobiotics by cytochrome P450 [PATH:ko00980]
C 00982 Drug metabolism - cytochrome P450 [PATH:ko00982]

C	00983 Drug metabolism - other enzymes [PATH:ko00983]
C	01000 Enzymes [BR:ko01000]
C	01001 Protein kinases [BR:ko01001]
C	01009 Protein phosphatase and associated proteins [BR:ko01009]
C	01002 Peptidases [BR:ko01002]
C	00199 Cytochrome P450 [BR:ko00199]

2. Pathway statistics of *Emticicia oligotrophica* DSM 17448

Complete pathways:

C	00010 Glycolysis / Gluconeogenesis [PATH:ko00010]
C	00020 Citrate cycle (TCA cycle) [PATH:ko00020]
C	00030 Pentose phosphate pathway [PATH:ko00030]
C	00040 Pentose and glucuronate interconversions [PATH:ko00040]
C	00190 Oxidative phosphorylation [PATH:ko00190]
C	00620 Pyruvate metabolism [PATH:ko00620]
C	00500 Starch and sucrose metabolism [PATH:ko00500]
C	00520 Amino sugar and nucleotide sugar metabolism [PATH:ko00520]
C	00250 Alanine, aspartate and glutamate metabolism [PATH:ko00250]
C	00061 Fatty acid biosynthesis [PATH:ko00061]
C	00561 Glycerolipid metabolism [PATH:ko00561]
C	00220 Arginine biosynthesis [PATH:ko00220]
C	00330 Arginine and proline metabolism [PATH:ko00330]
C	00340 Histidine metabolism [PATH:ko00340]
C	00400 Phenylalanine, tyrosine and tryptophan biosynthesis [PATH:ko00400]
C	00195 Photosynthesis [PATH:ko00195]
C	00230 Purine metabolism [PATH:ko00230]
C	00240 Pyrimidine metabolism [PATH:ko00240]
C	00540 Lipopolysaccharide biosynthesis [PATH:ko00540]
C	00550 Peptidoglycan biosynthesis [PATH:ko00550]
C	00740 Riboflavin metabolism [PATH:ko00740]
C	00750 Vitamin B6 metabolism [PATH:ko00750]
C	00780 Biotin metabolism [PATH:ko00780]
C	00760 Nicotinate and nicotinamide metabolism [PATH:ko00760]
C	00785 Lipoic acid metabolism [PATH:ko00785]
C	00720 Carbon fixation pathways in prokaryotes [PATH:ko00720]
C	00790 Folate biosynthesis [PATH:ko00790]
C	00770 Pantothenate and CoA biosynthesis [PATH:ko00770]
C	00640 Propanoate metabolism [PATH:ko00640]

Incomplete pathways:

C	00052 Galactose metabolism [PATH:ko00052]
C	00053 Ascorbate and aldarate metabolism [PATH:ko00053]
C	00051 Fructose and mannose metabolism [PATH:ko00051]
C	00630 Glyoxylate and dicarboxylate metabolism [PATH:ko00630]

C 00650 Butanoate metabolism [PATH:ko00650]
C 00660 C5-Branched dibasic acid metabolism [PATH:ko00660]
C 00562 Inositol phosphate metabolism [PATH:ko00562]
C 00680 Methane metabolism [PATH:ko00680]
C 00910 Nitrogen metabolism [PATH:ko00910]
C 00920 Sulfur metabolism [PATH:ko00920]
C 00072 Synthesis and degradation of ketone bodies [PATH:ko00072]
C 00564 Glycerophospholipid metabolism [PATH:ko00564]
C 00565 Ether lipid metabolism [PATH:ko00565]
C 00600 Sphingolipid metabolism [PATH:ko00600]
C 00590 Arachidonic acid metabolism [PATH:ko00590]
C 01040 Biosynthesis of unsaturated fatty acids [PATH:ko01040]
C 00260 Glycine, serine and threonine metabolism [PATH:ko00260]
C 00270 Cysteine and methionine metabolism [PATH:ko00270]
C 00280 Valine, leucine and isoleucine degradation [PATH:ko00280]
C 00290 Valine, leucine and isoleucine biosynthesis [PATH:ko00290]
C 00300 Lysine biosynthesis [PATH:ko00300]
C 00310 Lysine degradation [PATH:ko00310]
C 00350 Tyrosine metabolism [PATH:ko00350]
C 00360 Phenylalanine metabolism [PATH:ko00360]
C 00380 Tryptophan metabolism [PATH:ko00380]
C 00410 beta-Alanine metabolism [PATH:ko00410]
C 00430 Taurine and hypotaurine metabolism [PATH:ko00430]
C 00440 Phosphonate and phosphinate metabolism [PATH:ko00440]
C 00450 Selenocompound metabolism [PATH:ko00450]
C 00460 Cyanoamino acid metabolism [PATH:ko00460]
C 00471 D-Glutamine and D-glutamate metabolism [PATH:ko00471]
C 00472 D-Arginine and D-ornithine metabolism [PATH:ko00472]
C 00473 D-Alanine metabolism [PATH:ko00473]
C 00480 Glutathione metabolism [PATH:ko00480]
C 00510 N-Glycan biosynthesis [PATH:ko00510]
C 00513 Various types of N-glycan biosynthesis [PATH:ko00513]
C 00604 Glycosphingolipid biosynthesis - ganglio series [PATH:ko00604]
C 00511 Other glycan degradation [PATH:ko00511]
C 00571 Lipoarabinomannan (LAM) biosynthesis [PATH:ko00571]
C 00730 Thiamine metabolism [PATH:ko00730]
C 00785 Lipoic acid metabolism [PATH:ko00785]
C 00670 One carbon pool by folate [PATH:ko00670]
C 00130 Ubiquinone and other terpenoid-quinone biosynthesis [PATH:ko00130]
C 00900 Terpenoid backbone biosynthesis [PATH:ko00900]
C 00908 Zeatin biosynthesis [PATH:ko00908]
C 00903 Limonene and pinene degradation [PATH:ko00903]
C 00281 Geraniol degradation [PATH:ko00281]
C 01055 Biosynthesis of vancomycin group antibiotics [PATH:ko01055]

C 00940 Phenylpropanoid biosynthesis [PATH:ko00940]
C 00960 Tropane, piperidine and pyridine alkaloid biosynthesis [PATH:ko00960]
C 00966 Glucosinolate biosynthesis [PATH:ko00966]
C 00332 Carbapenem biosynthesis [PATH:ko00332]
C 00261 Monobactam biosynthesis [PATH:ko00261]
C 00524 Neomycin, kanamycin and gentamicin biosynthesis [PATH:ko00524]
C 00525 Acarbose and validamycin biosynthesis [PATH:ko00525]
C 00333 Prodigiosin biosyntheses [PATH:ko00333]
C 00362 Benzoate degradation [PATH:ko00362]
C 00627 Aminobenzoate degradation [PATH:ko00627]
C 00364 Fluorobenzoate degradation [PATH:ko00364]
C 00625 Chloroalkane and chloroalkene degradation [PATH:ko00625]
C 00361 Chlorocyclohexane and chlorobenzene degradation [PATH:ko00361]
C 00623 Toluene degradation [PATH:ko00623]
C 00622 Xylene degradation [PATH:ko00622]
C 00633 Nitrotoluene degradation [PATH:ko00633]
C 00642 Ethylbenzene degradation [PATH:ko00642]
C 00643 Styrene degradation [PATH:ko00643]
C 00983 Drug metabolism - other enzymes [PATH:ko00983]

Nonexistent pathways:

C 00196 Photosynthesis - antenna proteins [PATH:ko00196]
C 00710 Carbon fixation in photosynthetic organisms [PATH:ko00710]
C 00062 Fatty acid elongation [PATH:ko00062]
C 00071 Fatty acid degradation [PATH:ko00071]
C 00073 Cutin, suberine and wax biosynthesis [PATH:ko00073]
C 00100 Steroid biosynthesis [PATH:ko00100]
C 00120 Primary bile acid biosynthesis [PATH:ko00120]
C 00121 Secondary bile acid biosynthesis [PATH:ko00121]
C 00140 Steroid hormone biosynthesis [PATH:ko00140]
C 00591 Linoleic acid metabolism [PATH:ko00591]
C 00592 alpha-Linolenic acid metabolism [PATH:ko00592]
C 00512 Mucin type O-glycan biosynthesis [PATH:ko00512]
C 00515 Mannose type O-glycan biosyntheis [PATH:ko00515]
C 00514 Other types of O-glycan biosynthesis [PATH:ko00514]
C 00532 Glycosaminoglycan biosynthesis - chondroitin sulfate / dermatan sulfate [PATH:ko00532]
C 00534 Glycosaminoglycan biosynthesis - heparan sulfate / heparin [PATH:ko00534]
C 00533 Glycosaminoglycan biosynthesis - keratan sulfate [PATH:ko00533]
C 00531 Glycosaminoglycan degradation [PATH:ko00531]
C 00563 Glycosylphosphatidylinositol(GPI)-anchor biosynthesis [PATH:ko00563]
C 00601 Glycosphingolipid biosynthesis - lacto and neolacto series [PATH:ko00601]
C 00603 Glycosphingolipid biosynthesis - globo and isoglobo series [PATH:ko00603]
C 00830 Retinol metabolism [PATH:ko00830]

C 00860 Porphyrin and chlorophyll metabolism [PATH:ko00860]
C 00902 Monoterpenoid biosynthesis [PATH:ko00902]
C 00909 Sesquiterpenoid and triterpenoid biosynthesis [PATH:ko00909]
C 00904 Diterpenoid biosynthesis [PATH:ko00904]
C 00906 Carotenoid biosynthesis [PATH:ko00906]
C 00902 Monoterpenoid biosynthesis [PATH:ko00902]
C 00909 Sesquiterpenoid and triterpenoid biosynthesis [PATH:ko00909]
C 00904 Diterpenoid biosynthesis [PATH:ko00904]
C 00906 Carotenoid biosynthesis [PATH:ko00906]
C 01052 Type I polyketide structures [PATH:ko01052]
C 00522 Biosynthesis of 12-, 14- and 16-membered macrolides [PATH:ko00522]
C 01051 Biosynthesis of ansamycins [PATH:ko01051]
C 01059 Biosynthesis of enediyne antibiotics [PATH:ko01059]
C 01056 Biosynthesis of type II polyketide backbone [PATH:ko01056]
C 01057 Biosynthesis of type II polyketide products [PATH:ko01057]
C 00253 Tetracycline biosynthesis [PATH:ko00253]
C 00523 Polyketide sugar unit biosynthesis [PATH:ko00523]
C 01054 Nonribosomal peptide structures [PATH:ko01054]
C 01053 Biosynthesis of siderophore group nonribosomal peptides [PATH:ko01053]
C 00945 Stilbenoid, diarylheptanoid and gingerol biosynthesis [PATH:ko00945]
C 00941 Flavonoid biosynthesis [PATH:ko00941]
C 00944 Flavone and flavonol biosynthesis [PATH:ko00944]
C 00942 Anthocyanin biosynthesis [PATH:ko00942]
C 00943 Isoflavonoid biosynthesis [PATH:ko00943]
C 00901 Indole alkaloid biosynthesis [PATH:ko00901]
C 00403 Indole diterpene alkaloid biosynthesis [PATH:ko00403]
C 00950 Isoquinoline alkaloid biosynthesis [PATH:ko00950]
C 01058 Acridone alkaloid biosynthesis [PATH:ko01058]
C 00232 Caffeine metabolism [PATH:ko00232]
C 00965 Betalain biosynthesis [PATH:ko00965]
C 00402 Benzoxazinoid biosynthesis [PATH:ko00402]
C 00311 Penicillin and cephalosporin biosynthesis [PATH:ko00311]
C 00331 Clavulanic acid biosynthesis [PATH:ko00331]
C 00521 Streptomycin biosynthesis [PATH:ko00521]
C 00231 Puromycin biosynthesis [PATH:ko00231]
C 00401 Novobiocin biosynthesis [PATH:ko00401]
C 00404 Staurosporine biosynthesis [PATH:ko00404]
C 00405 Phenazine biosynthesis [PATH:ko00405]
C 00254 Aflatoxin biosynthesis [PATH:ko00254]
C 00999 Biosynthesis of secondary metabolites - unclassified [PATH:ko00999]
C 00791 Atrazine degradation [PATH:ko00791]
C 00930 Caprolactam degradation [PATH:ko00930]
C 00363 Bisphenol degradation [PATH:ko00363]
C 00621 Dioxin degradation [PATH:ko00621]

C	00626 Naphthalene degradation [PATH:ko00626]
C	00624 Polycyclic aromatic hydrocarbon degradation [PATH:ko00624]
C	00365 Furfural degradation [PATH:ko00365]
C	00984 Steroid degradation [PATH:ko00984]
C	01003 Glycosyltransferases [BR:ko01003]
C	00980 Metabolism of xenobiotics by cytochrome P450 [PATH:ko00980]
C	00982 Drug metabolism - cytochrome P450 [PATH:ko00982]
C	00536 Glycosaminoglycan binding proteins [BR:ko00536]
C	00535 Proteoglycans [BR:ko00535]
C	00351 1,1,1-Trichloro-2,2-bis(4-chlorophenyl)ethane (DDT) degradation [PATH:ko00351]
C	01008 Polyketide biosynthesis proteins [BR:ko01008]
C	01002 Peptidases [BR:ko01002]

3. Pathway statistics of *Leadbetterlla byssophila* DSM 17132
Complete Pathways:

C	00010 Glycolysis / Gluconeogenesis [PATH:ko00010]
C	00020 Citrate cycle (TCA cycle) [PATH:ko00020]
C	00030 Pentose phosphate pathway [PATH:ko00030]
C	00040 Pentose and glucuronate interconversions [PATH:ko00040]
C	00620 Pyruvate metabolism [PATH:ko00620]
C	00190 Oxidative phosphorylation [PATH:ko00190]
C	00250 Alanine, aspartate and glutamate metabolism [PATH:ko00250]
C	00500 Starch and sucrose metabolism [PATH:ko00500]
C	00061 Fatty acid biosynthesis [PATH:ko00061]
C	00561 Glycerolipid metabolism [PATH:ko00561]
C	00220 Arginine biosynthesis [PATH:ko00220]
C	00330 Arginine and proline metabolism [PATH:ko00330]
C	00340 Histidine metabolism [PATH:ko00340]
C	00400 Phenylalanine, tyrosine and tryptophan biosynthesis [PATH:ko00400]
C	00195 Photosynthesis [PATH:ko00195]
C	00720 Carbon fixation pathways in prokaryotes [PATH:ko00720]
C	00230 Purine metabolism [PATH:ko00230]
C	00240 Pyrimidine metabolism [PATH:ko00240]
C	00290 Valine, leucine and isoleucine biosynthesis [PATH:ko00290]
C	00540 Lipopolysaccharide biosynthesis [PATH:ko00540]
C	00550 Peptidoglycan biosynthesis [PATH:ko00550]
C	00740 Riboflavin metabolism [PATH:ko00740]
C	00750 Vitamin B6 metabolism [PATH:ko00750]
C	00780 Biotin metabolism [PATH:ko00780]
C	00770 Pantothenate and CoA biosynthesis [PATH:ko00770]
C	00790 Folate biosynthesis [PATH:ko00790]
C	00760 Nicotinate and nicotinamide metabolism [PATH:ko00760]
C	00785 Lipoic acid metabolism [PATH:ko00785]

Incomplete pathways:

C 00051 Fructose and mannose metabolism [PATH:ko00051]
C 00520 Amino sugar and nucleotide sugar metabolism [PATH:ko00520]
C 00052 Galactose metabolism [PATH:ko00052]
C 00640 Propanoate metabolism [PATH:ko00640]
C 00310 Lysine degradation [PATH:ko00310]
C 00660 C5-Branched dibasic acid metabolism [PATH:ko00660]
C 00650 Butanoate metabolism [PATH:ko00650]
C 00562 Inositol phosphate metabolism [PATH:ko00562]
C 00680 Methane metabolism [PATH:ko00680]
C 00680 Methane metabolism [PATH:ko00680]
C 00920 Sulfur metabolism [PATH:ko00920]
C 00071 Fatty acid degradation [PATH:ko00071]
C 00072 Synthesis and degradation of ketone bodies [PATH:ko00072]
C 01040 Biosynthesis of unsaturated fatty acids [PATH:ko01040]
C 00350 Tyrosine metabolism [PATH:ko00350]
C 00360 Phenylalanine metabolism [PATH:ko00360]
C 00380 Tryptophan metabolism [PATH:ko00380]
C 00410 beta-Alanine metabolism [PATH:ko00410]
C 00430 Taurine and hypotaurine metabolism [PATH:ko00430]
C 00053 Ascorbate and aldarate metabolism [PATH:ko00053]
C 00630 Glyoxylate and dicarboxylate metabolism [PATH:ko00630]
C 00564 Glycerophospholipid metabolism [PATH:ko00564]
C 00565 Ether lipid metabolism [PATH:ko00565]
C 00600 Sphingolipid metabolism [PATH:ko00600]
C 00590 Arachidonic acid metabolism [PATH:ko00590]
C 00260 Glycine, serine and threonine metabolism [PATH:ko00260]
C 00270 Cysteine and methionine metabolism [PATH:ko00270]
C 00280 Valine, leucine and isoleucine degradation [PATH:ko00280]
C 00440 Phosphonate and phosphinate metabolism [PATH:ko00440]
C 00450 Selenocompound metabolism [PATH:ko00450]
C 00460 Cyanoamino acid metabolism [PATH:ko00460]
C 00471 D-Glutamine and D-glutamate metabolism [PATH:ko00471]
C 00480 Glutathione metabolism [PATH:ko00480]
C 00510 N-Glycan biosynthesis [PATH:ko00510]
C 00513 Various types of N-glycan biosynthesis [PATH:ko00513]
C 00604 Glycosphingolipid biosynthesis - ganglio series [PATH:ko00604]
C 00511 Other glycan degradation [PATH:ko00511]
C 00571 Lipoarabinomannan (LAM) biosynthesis [PATH:ko00571]
C 00730 Thiamine metabolism [PATH:ko00730]
C 00670 One carbon pool by folate [PATH:ko00670]
C 00830 Retinol metabolism [PATH:ko00830]
C 00860 Porphyrin and chlorophyll metabolism [PATH:ko00860]
C 00130 Ubiquinone and other terpenoid-quinone biosynthesis [PATH:ko00130]

C 00900 Terpenoid backbone biosynthesis [PATH:ko00900]
81]
C 00908 Zeatin biosynthesis [PATH:ko00908]
C 00903 Limonene and pinene degradation [PATH:ko00903]
C 00281 Geraniol degradation [PATH:ko00281]
C 01055 Biosynthesis of vancomycin group antibiotics [PATH:ko01055]
C 00940 Phenylpropanoid biosynthesis [PATH:ko00940]
C 00960 Tropane, piperidine and pyridine alkaloid biosynthesis [PATH:ko00960]
C 00966 Glucosinolate biosynthesis [PATH:ko00966]
C 00332 Carbapenem biosynthesis [PATH:ko00332]
C 00261 Monobactam biosynthesis [PATH:ko00261]
C 00524 Neomycin, kanamycin and gentamicin biosynthesis [PATH:ko00524]
C 00525 Acarbose and validamycin biosynthesis [PATH:ko00525]
C 00333 Prodigiosin biosyntheses [PATH:ko00333]
C 00362 Benzoate degradation [PATH:ko00362]
C 00625 Chloroalkane and chloroalkene degradation [PATH:ko00625]
C 00361 Chlorocyclohexane and chlorobenzene degradation [PATH:ko00361]
C 00623 Toluene degradation [PATH:ko00623]
C 00643 Styrene degradation [PATH:ko00643]

Nonexistent Pathways:
C 00196 Photosynthesis - antenna proteins [PATH:ko00196]
C 00710 Carbon fixation in photosynthetic organisms [PATH:ko00710]
C 00062 Fatty acid elongation [PATH:ko00062]
C 00073 Cutin, suberine and wax biosynthesis [PATH:ko00073]
C 00100 Steroid biosynthesis [PATH:ko00100]
C 00120 Primary bile acid biosynthesis [PATH:ko00120]
C 00121 Secondary bile acid biosynthesis [PATH:ko00121]
C 00140 Steroid hormone biosynthesis [PATH:ko00140]
C 00591 Linoleic acid metabolism [PATH:ko00591]
C 00592 alpha-Linolenic acid metabolism [PATH:ko00592]
C 00472 D-Arginine and D-ornithine metabolism [PATH:ko00472]
C 00473 D-Alanine metabolism [PATH:ko00473]
C 00512 Mucin type O-glycan biosynthesis [PATH:ko00512]
C 00515 Mannose type O-glycan biosyntheis [PATH:ko00515]
C 00514 Other types of O-glycan biosynthesis [PATH:ko00514]
C 00532 Glycosaminoglycan biosynthesis - chondroitin sulfate / dermatan sulfate [PATH:ko00532]
C 00534 Glycosaminoglycan biosynthesis - heparan sulfate / heparin [PATH:ko00534]
C 00533 Glycosaminoglycan biosynthesis - keratan sulfate [PATH:ko00533]
C 00531 Glycosaminoglycan degradation [PATH:ko00531]
C 00563 Glycosylphosphatidylinositol(GPI)-anchor biosynthesis [PATH:ko00563]
C 00601 Glycosphingolipid biosynthesis - lacto and neolacto series [PATH:ko00601]
C 00603 Glycosphingolipid biosynthesis - globo and isoglobo series [PATH:ko00603]

C 00902 Monoterpenoid biosynthesis [PATH:ko00902]
C 00909 Sesquiterpenoid and triterpenoid biosynthesis [PATH:ko00909]
C 00904 Diterpenoid biosynthesis [PATH:ko00904]
C 00906 Carotenoid biosynthesis [PATH:ko00906]
C 00905 Brassinosteroid biosynthesis [PATH:ko00905]
C 00981 Insect hormone biosynthesis [PATH:ko009]
C 01052 Type I polyketide structures [PATH:ko01052]
C 00522 Biosynthesis of 12-, 14- and 16-membered macrolides [PATH:ko00522]
C 01051 Biosynthesis of ansamycins [PATH:ko01051]
C 01059 Biosynthesis of enediyne antibiotics [PATH:ko01059]
C 01056 Biosynthesis of type II polyketide backbone [PATH:ko01056]
C 01057 Biosynthesis of type II polyketide products [PATH:ko01057]
C 00253 Tetracycline biosynthesis [PATH:ko00253]
C 00523 Polyketide sugar unit biosynthesis [PATH:ko00523]
C 01054 Nonribosomal peptide structures [PATH:ko01054]
C 01053 Biosynthesis of siderophore group nonribosomal peptides [PATH:ko01053]
C 00945 Stilbenoid, diarylheptanoid and gingerol biosynthesis [PATH:ko00945]
C 00941 Flavonoid biosynthesis [PATH:ko00941]
C 00944 Flavone and flavonol biosynthesis [PATH:ko00944]
C 00942 Anthocyanin biosynthesis [PATH:ko00942]
C 00943 Isoflavonoid biosynthesis [PATH:ko00943]
C 00901 Indole alkaloid biosynthesis [PATH:ko00901]
C 00403 Indole diterpene alkaloid biosynthesis [PATH:ko00403]
C 00950 Isoquinoline alkaloid biosynthesis [PATH:ko00950]
C 01058 Acridone alkaloid biosynthesis [PATH:ko01058]
C 00232 Caffeine metabolism [PATH:ko00232]
C 00965 Betalain biosynthesis [PATH:ko00965]
C 00402 Benzoxazinoid biosynthesis [PATH:ko00402]
C 00311 Penicillin and cephalosporin biosynthesis [PATH:ko00311]
C 00331 Clavulanic acid biosynthesis [PATH:ko00331]
C 00521 Streptomycin biosynthesis [PATH:ko00521]
C 00231 Puromycin biosynthesis [PATH:ko00231]
C 00401 Novobiocin biosynthesis [PATH:ko00401]
C 00404 Staurosporine biosynthesis [PATH:ko00404]
C 00405 Phenazine biosynthesis [PATH:ko00405]
C 00199 Cytochrome P450 [BR:ko00199]
C 00254 Aflatoxin biosynthesis [PATH:ko00254]
C 00999 Biosynthesis of secondary metabolites - unclassified [PATH:ko00999]
C 00627 Aminobenzoate degradation [PATH:ko00627]
C 00364 Fluorobenzoate degradation [PATH:ko00364]
C 00622 Xylene degradation [PATH:ko00622]
C 00535 Proteoglycans [BR:ko00535]
C 01003 Glycosyltransferases [BR:ko01003]
C 00536 Glycosaminoglycan binding proteins [BR:ko00536]

C 00351 1,1,1-Trichloro-2,2-bis(4-chlorophenyl)ethane (DDT) degradation [PATH:ko00351]
C 01008 Polyketide biosynthesis proteins [BR:ko01008]
C 01006 Prenyltransferases [BR:ko01006]
C 00910 Nitrogen metabolism [PATH:ko00910]
C 01007 Amino acid related enzymes [BR:ko01007]
C 00633 Nitrotoluene degradation [PATH:ko00633]
C 00642 Ethylbenzene degradation [PATH:ko00642]
C 00791 Atrazine degradation [PATH:ko00791]
C 00930 Caprolactam degradation [PATH:ko00930]
C 00363 Bisphenol degradation [PATH:ko00363]
C 00621 Dioxin degradation [PATH:ko00621]
C 00626 Naphthalene degradation [PATH:ko00626]
C 00624 Polycyclic aromatic hydrocarbon degradation [PATH:ko00624]
C 00365 Furfural degradation [PATH:ko00365]
C 00984 Steroid degradation [PATH:ko00984]
C 00980 Metabolism of xenobiotics by cytochrome P450 [PATH:ko00980]
C 00982 Drug metabolism - cytochrome P450 [PATH:ko00982]
C 00983 Drug metabolism - other enzymes [PATH:ko00983]
C 01002 Peptidases [BR:ko01002]
